# Modeling the response to interleukin‐21 to inform natural killer cell immunotherapy

**DOI:** 10.1111/imcb.12848

**Published:** 2025-01-25

**Authors:** Indrani Nayak, Rosalba Biondo, William C Stewart, Rebecca J Fulton, Nina Möker, Congcong Zhang, Salim I Khakoo, Jayajit Das

**Affiliations:** ^1^ Steve and Cindy Rasmussen Institute for Genomic Medicine Abigail Wexner Research Institute, Nationwide Children's Hospital Columbus OH USA; ^2^ School of Clinical and Experimental Sciences University of Southampton Southampton UK; ^3^ GIG Statistical Consulting, LLC Columbus OH USA; ^4^ Miltenyi Biotec B.V. & Co. KG Bergisch Gladbach Germany; ^5^ Biomedical Sciences Graduate Program The Ohio State University Columbus OH USA; ^6^ Department of Pediatrics The Ohio State University Columbus OH USA; ^7^ Pelotonia Institute for Immuno‐Oncology The Ohio State University Columbus OH USA; ^8^ The Biophysics Graduate Program The Ohio State University Columbus OH USA

**Keywords:** cytokines, linear regression, NK cells, predictive model, proliferation, STAT

## Abstract

Natural killer (NK) cells are emerging agents for cancer therapy. Several different cytokines are used to generate NK cells for adoptive immunotherapy including interleukin (IL)‐2, IL‐12, IL‐15 and IL‐18 in solution, and membrane‐bound IL‐21. These cytokines drive NK cell activation through the integration of signal transducers and activators of transcription (STAT) and nuclear factor‐kappa B (NF‐κB) pathways, which overlap and synergize, making it challenging to predict optimal cytokine combinations for both proliferation and cytotoxicity. We integrated functional assays for NK cells cultured in a variety of cytokine combinations with mathematical modeling using feature selection and mechanistic regression models. Our regression model successfully predicts NK cell proliferation for different cytokine combinations and indicates synergy of activated STATs and NF‐κB transcription factors between priming and post‐priming phases. The use of IL‐21 in solution in the priming of NK cell culture resulted in an improved NK cell proliferation, without compromising cytotoxicity potential or interferon gamma secretion against hepatocellular carcinoma cell lines. Our work provides an integrative framework for interrogating NK cell proliferation and activation for cancer immunotherapy.
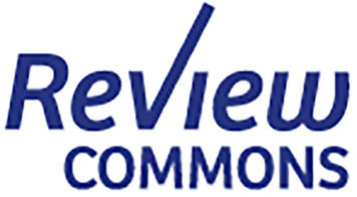

## Graphical abstract

In this study, we investigated multiple cytokine combinations to boost natural killer (NK) cell proliferation as well as effector functions of proliferated NK cells against hepatocellular carcinoma as a potential immunotherapy application. Using a combination of *in vitro* experiments and *in silico* regression modeling incorporating the complex interplay between signal transducers and activators of transcription (STAT) and nuclear factor‐kappa B (NF‐κB) induced by the cytokine cocktail, we find that the inclusion of IL‐21 in cytokine mixtures significantly enhances NK cell proliferation and cytotoxicity against hepatocellular carcinoma cell lines.

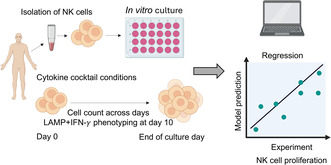

## INTRODUCTION

Natural killer (NK) cells are being increasingly used as an adoptive immunotherapy against tumors. One key challenge in NK cell immunotherapy is the ability to generate sufficient numbers of activated NK cells to be an effective therapeutic. Expansion of NK cells *in vitro* using specific combinations of cytokines is a promising approach to generate an adequate quality and quantity of NK cells.^1^, ^2^, ^3^, ^4^, ^5^ NK cells express multiple cytokine receptors for binding to type 1 [interleukin (IL)‐2/4/12/15/21], type 2 [such as interferon gamma (IFNγ)] and IL‐1 (such as IL‐18) family cytokines.^6^, ^7^ Thus, there are many potential combinations of cytokines that could be used to expand NK cells. A widely used protocol to induce proliferation and maturation of NK cells *in vitro* is to prestimulate NK cells with a combination of cytokines for a short duration, up to 16 h (priming) followed by culture over days where fewer cytokines are used.^1^ *Ex vivo* expansion using IL‐12/15/18 priming to generate long‐lived memory NK cells is a promising area of research with clinical benefit obtained in early phase studies.^1^, ^8^, ^9^ IL‐21 is a pleiotropic cytokine that is involved in the activation of NK and CD8^+^ T cells and is specifically associated with tissue‐resident NK cells.^10^ IL‐21 has been shown to induce excellent expansion of NK cells *ex vivo* using feeder cell lines expressing membrane‐bound IL‐21, which appears to mitigate the senescence associated with other cytokine regimens.^11^ However, the use of feeder cell lines has additional regulatory issues relating to the provenance of the cell lines used.^12^ Combining IL‐21 with preexisting regimens using soluble cytokines provides an alternative strategy to NK cell expansion.

Selecting the correct combinations of cytokines to obtain an NK cell population with a desired response, such as large population size and/or greater cytotoxicity against specific target cells, is a key challenge. Although the effects of single cytokines on NK cells is well understood, the difficulty in choosing regimens using multiple cytokines arises as a result of our rudimentary understanding of how the different combinations of cytokines combine to stimulate NK cells and induce genes that regulate NK cell proliferation, expression of specific NK cell receptors and NK cell maturation. NK cells are activated by the stimulation of cell surface and cytokine receptors in a combinatorial manner. Signals from different combinations of receptors can synergize to enhance NK cell proliferation and activation.

Stimulation by the type 1 and 2 family of cytokines leads to the phosphorylation of seven signal transducer and activator of transcription (STAT) proteins and stimulation by IL‐1 family of cytokines activates nuclear factor‐kappa B (NF‐κB) transcription factor. A cytokine can give rise to the activation of multiple STAT proteins or NF‐κB; here we denote the STAT proteins/NF‐κB molecules that are preferentially activated by a cytokine as *primary* STATs/NF‐κB, while those activated to a lesser extent are referred as *secondary* STATs/NF‐κB.^6^, ^7^, ^13^ These proteins translocate to the nucleus upon phosphorylation and induce specific gene expressions by binding to promoters/enhancers motifs in the DNA.^13^ However, there is a substantial overlap between the activated STAT proteins that are phosphorylated as a result of different cytokine stimulation; for example, STAT1, STAT3 and STAT4 can be phosphorylated by either IL‐2 or IL‐12 stimulation.^6^, ^7^, ^13^ In addition, the STAT proteins can synergize or antagonize as they induce transcription.^6^ Furthermore, NK cells obtained from different donors can respond differently to the same combination of cytokines. Therefore, relating a combination of cytokines to a specific NK cell response such as proliferation across donors can be challenging. While mechanistic computational models based on ordinary differential equations have been developed to describe NK‐, T‐ and B‐cell proliferation under the influence of a variety of cytokines including IL‐2 and/or IL‐15,^14^, ^15^, ^16^, ^17^, ^18^ it is challenging to extend such models to include priming and post‐priming by a cocktail of cytokines.

Machine learning–based or mechanistic ordinary differential equation and stochastic simulation–based models have been explored in relating cytokines with predictive outcomes of pathogenesis, or T‐cell activation^19^, ^20^, ^21^, ^22^, ^23^ and kinetics of phosphorylated STAT proteins and gene expression responses in bone marrow–derived macrophages^24^ and dendritic cells^25^ treated with cytokines over several hours to few days. However, to date, the roles of synergy/antagonism between multiple cytokines in regulating NK cell proliferation remain unexplored. By including the effect of synergy/antagonism between STAT and NF‐κB transcription factors induced by multiple cytokines, we address this challenge by developing a predictive framework combining longitudinal measurement of NK cell populations, regression model with least absolute shrinkage and selection operator (LASSO) regularization^26^ and a filter‐style feature selection algorithm (RRelief),^27^ commonly used for guiding machine learning methods, that can predict fold expansions in NK cell population using different cytokine combinations.

## RESULTS

### IL‐21 induces improved proliferation of NK cells

**Figure 1:**
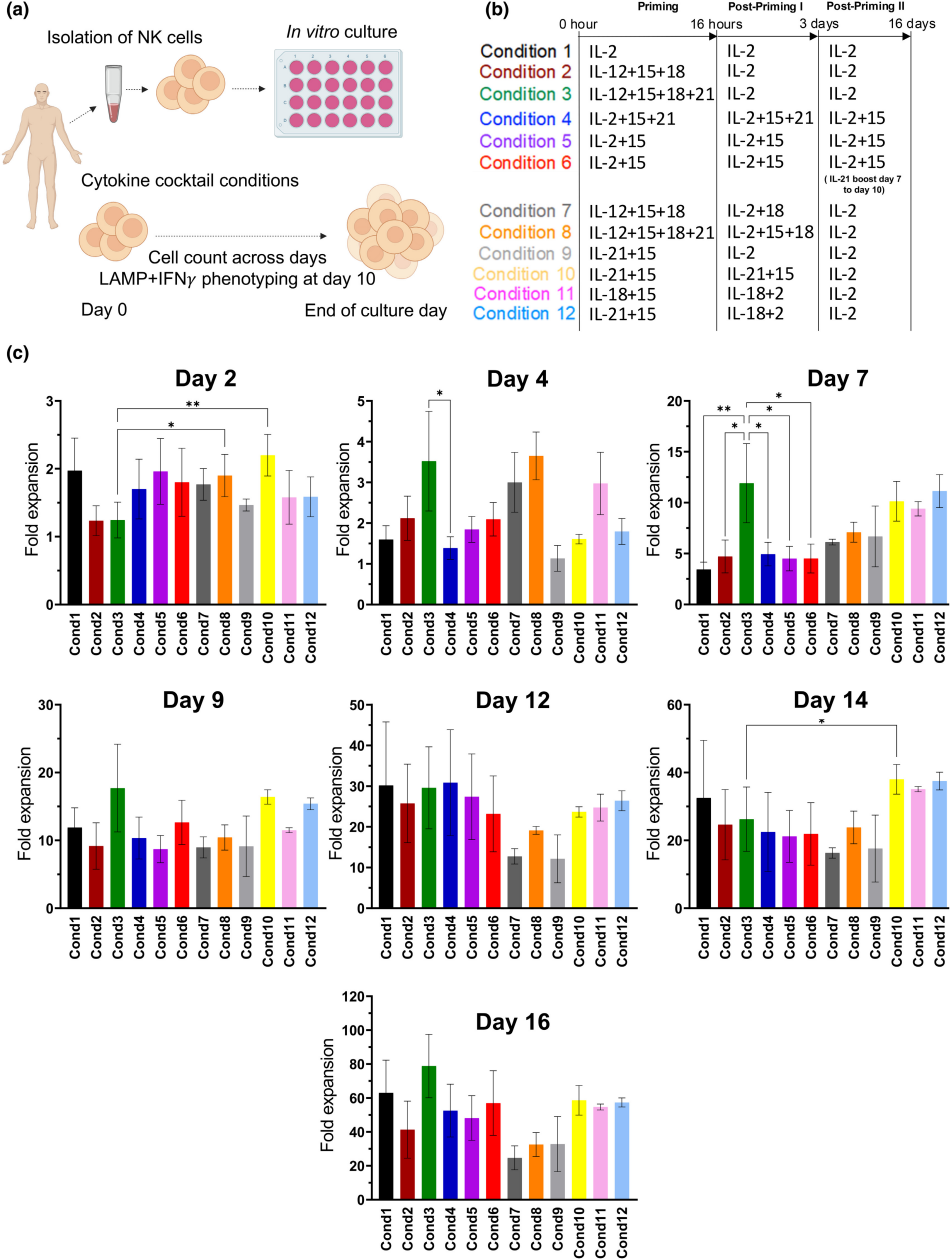
Testing different cytokine conditions for natural killer (NK) cell proliferation. **(a)** NK cells were isolated from the peripheral blood of 17 healthy donors and cultured with different combinations of cytokines with fresh media replenished every 2–3 days. Cells were counted at days 0, 2, 4, 7, 9, 12, 14 and 16. Lysosome‐associated membrane protein (LAMP) or CD107a and IFNγ expression were measured on day 10. **(b)** The different combinations of cytokines used to culture NK cells. For condition 6, interleukin (IL)‐21 boost indicates the addition of IL‐21 for 3 days of culture. IL‐21 was added on day 7 of culture, until day 10, after which the media was changed and the IL‐2 + 15 cytokine combination was used. **(c)** Proliferation of NK cells treated with the regimens as illustrated in **(b)**. Ten donors were treated with conditions 1–6, 4 donors with conditions 7 and 8 and 3 donors with conditions 9–12. Data are shown as mean ± standard error of the mean (*P ≤ 0.05, **P ≤ 0.01). Data were analyzed by one‐way ANOVA and Dunnett's multiple comparison test.

To investigate the effects of different cytokines on the proliferation of NK cells, NK cells were purified from 17 donors and treated with 12 different combinations of IL‐2, IL‐12, IL‐15, IL‐18 and IL‐21 (Figure 1a, b). Ten donors (donors 1–10) were treated with cytokine conditions 1–6, four donors (donors 11–14) were treated with cytokine conditions 7 and 8, and three donors (donors 15–17) were treated with conditions 9–12 (Supplementary table 1a). The experimental design involved a priming step of 16 h, followed by a post‐priming step consisting of a different cytokine combination for up to 3 days (denoted as post‐priming I or PP‐I), and finally a maintenance regimen of IL‐2 alone or IL‐2 in combination with IL‐15 (denoted as post‐priming II or PP‐II), as shown in Figure 1b. Cells were cultured for up to 16 days and the average NK cell fold expansion was measured across donors for each cytokine cocktail condition on days 2, 4, 7, 9, 12, 14 and 16 (Figure 1c and Supplementary table 2b). IL‐21 exhibited an enhanced proliferative effect as early as day 4 of culture. This effect was further amplified when IL‐21 was used in the culture combination condition 3, resulting in a greater than 10‐fold proliferation by day 7 (*P* < 0.05 or *P* < 0.01; Figure 1c). By day 9 under all conditions, NK cells had proliferated between 10‐ and 15‐fold, with the IL‐21 priming regimens, such as condition 3, giving better early proliferation as compared with condition 2. The effects of IL‐21 showed a general positive trend by this stage, but it was less evident in the later stages of culture. However, there was a substantial donor heterogeneity within these responses, with one donor having a ~70‐fold proliferation with condition 3, which was approximately threefold higher than any other donor (Supplementary table 1a).

**Figure 2:**
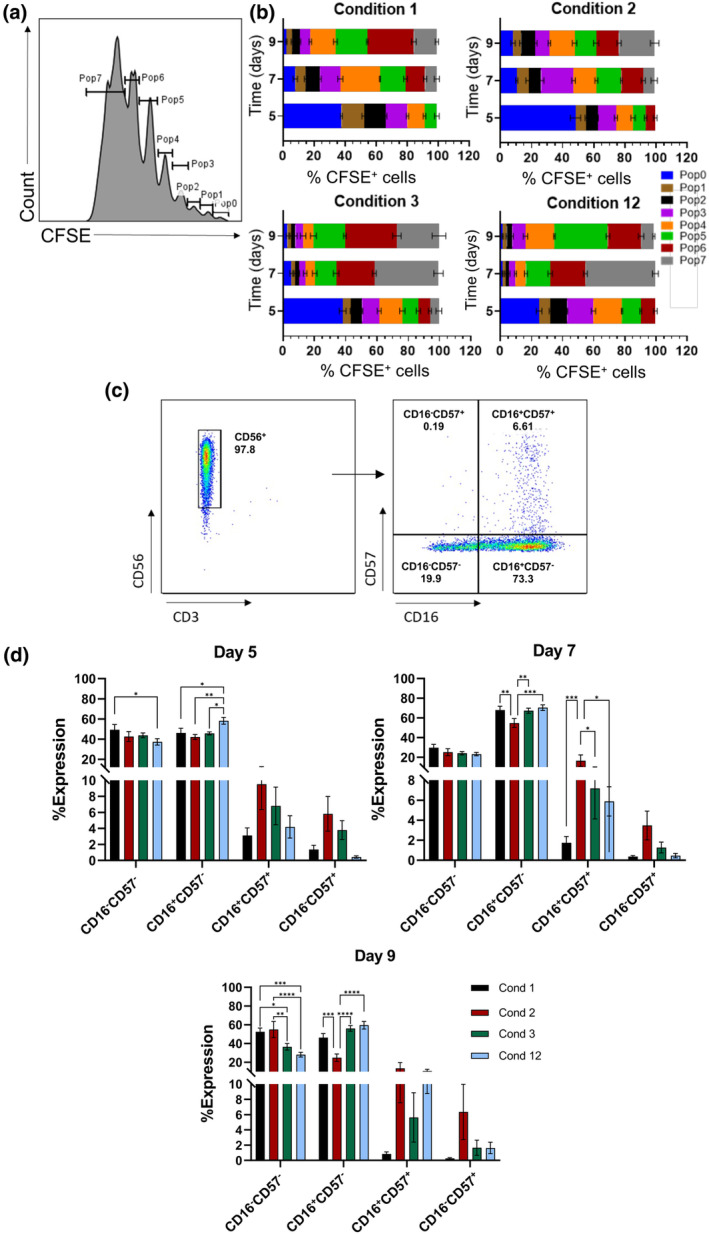
Proliferation and maturation of natural killer (NK) cell subpopulations. NK cells from 4 donors were stained with CellTrace carboxyfluorescein succinimidyl ester (CFSE); cultured with condition 1, condition 2, condition 3 and condition 12; and assessed by flow cytometry on days 5, 7 and 9 of *in vitro* culture. **(a)** Representative example of gating strategy. The whole NK cell population was gated on each cell division, according to the levels of fluorescence of CFSE. This was used to determine the size of each subpopulation, plotted in **(b)**. **(b)** Proliferation of each NK subpopulation, based on their CFSE percentage. The width of each color segment reflects the proportion of the individual populations in the entire CFSE^+^ cell population. Each division is marked by a difference in CFSE retention by the cells, marking the different populations (pop0 to pop7) within the whole heterogenous cell pool. Data are shown as mean ± standard error of the mean (*n* = 4). **(c)** Representative example of gating strategy. CD56^+^ NK cells were analyzed for the expression of CD16 and CD57 by flow cytometry. **(d)** Proportions of NK subpopulations according to the expression of CD16 and CD57, on days 5, 7 and 9, as shown in the gating strategy in **(c)**. Data are shown as mean ± standard error of the mean (*n* = 4), analyzed by two‐way ANOVA with Tukey's multiple comparison test (*P ≤ 0.05, **P ≤ 0.01, ***P ≤ 0.001, ****P ≤ 0.0001).

To investigate the role of IL‐21 in NK cell proliferation further, we selected the two regimens that contained IL‐21 only in the priming phase (conditions 3 and 12). We used carboxyfluorescein succinimidyl ester (CFSE) staining to determine the rate of cell division as compared with a control condition IL‐2 alone or IL‐12/15/18 priming (Figure 2a, b). Consistent with our initial observations (Figure 1c), the difference in proliferation was most clearly seen on day 7 (gray bars in Figure 2b) for IL‐21–containing regimens (conditions 3 and 12), compared with the control conditions (conditions 1 and 2). A higher proportion of NK cells had undergone seven cell divisions by day 7 compared with day 9, for both IL‐21–containing condition (population 7 as gray bars in Figure 2b). The percentage of CFSE–positive NK cells from population 7 decreased from a mean of 40% on day 7 to 25% on day 9 in condition 3, and from 45% on day 7 to 10% in condition 12 (Figure 2b). The highest relative decrease in the proportion of NK cell undergoing seven cell divisions was showed by condition 12. This condition had IL‐21 and IL‐15 in the priming step, as opposed to conditions with IL‐12/15/18 plus IL‐21, that is, inclusion of IL‐21 with the full set of cytokines used in the clinic to induce memory NK cells (population 7; Figure 2b). The greatest loss of NK cells was found in condition 12, which had IL‐21 and IL‐15 in the priming step, as opposed to conditions with IL‐12/15/18 plus IL‐21, that is, inclusion of IL‐21 with the full set of cytokines used in the clinic to induce memory NK cells (population 7, Figure 2b).

To assess the maturation status of proliferating NK cell subsets, the expression of CD16 and CD57 was evaluated on days 5, 7 and 9. CD16 is a well‐established marker of mature NK cells, whereas CD57 indicates terminal differentiation, and sometimes exhaustion (Figure 2c, d). The gating strategy for this analysis is shown in Figure 2c. Overall, the NK cells were predominantly of a CD16^+^CD57^−^ phenotype, which was greater in IL‐21–containing conditions (conditions 3 and 12; Figure 2d). On day 5, the CD16^+^CD57^−^ subpopulation comprised 40–50% of the NK population in condition 12, and this was greater than the two non–IL‐21 containing regimens (*P* < 0.05 or *P* < 0.01; Figure 2d). By day 7, over 60% of the NK cell population showed a phenotype which was predominantly CD16^+^CD57^−^ in all conditions but was higher in the IL‐21–containing regimens than in the IL‐12/15/18 group. In addition, there were relatively fewer exhausted CD16^+^CD57^+^ or terminally mature CD16^−^CD57^+^ NK cells in the IL‐21–containing regimens (*P* < 0.05; Figure 2d). By day 9 of culture, approximately 20% of the CD56^+^ NK cell population had a CD16^−^CD57^−^ phenotype in the presence of IL‐21 (conditions 3 and 12), compared with over 40% when IL‐21 is not present (conditions 1 and 2; day 9; Figure 2d). Thus, IL‐21 supports the maintenance of a key mature cytotoxic subpopulation, with the phenotype CD16^+^CD57^−^, which might be more susceptible to cytokine stimulation.^28^

### IL21–STAT3 signaling determines proliferation

**Figure 3:**
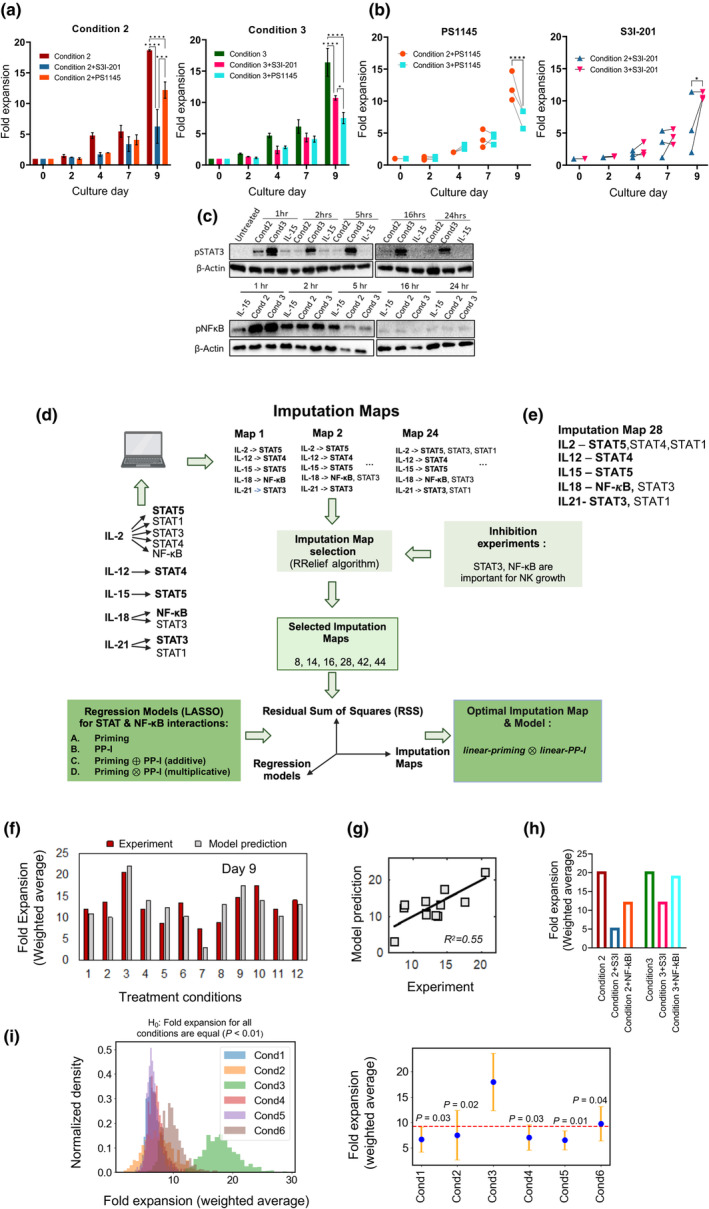
Developing and testing a model for natural killer (NK) cell proliferation: interleukin (IL)‐21 sustains early proliferation *in vitro*. **(a, b)** Isolated NK cells from three donors were cultured with the cytokine combination condition 2: IL‐12 + 15 + 18 priming for 16 h, then IL‐2 only, and condition 3: IL‐12 + 15 + 18 + 21 priming for 16 h, and then IL‐2 only. During the priming stage, NK cells were treated with either 75 μM S3I‐201 [signal transducers and activators of transcription 3 (STAT3) inhibitor] or 10 μM PS1145 [nuclear factor‐kappa B (NF‐κB) inhibitor]. Cells were then washed and cultured only with IL‐2. Data are shown as mean ± standard error of the mean. The fold expansions for the two conditions are shown in **(a)**, and the comparisons for the two conditions are shown in **(b)**. Data were analyzed by two‐way ANOVA and Tukey's multiple comparison test (*P ≤ 0.05, ***P ≤ 0.001, ****P ≤ 0.0001). **(c)** Isolated NK cells were cultured with condition 2, condition 3, or IL‐15 only (1 ng mL^−1^) at the indicated time points, and then pSTAT3 and pNF‐κB along with β‐actin protein levels were detected by immunoblotting. **(d)** Schematic depiction of the construction of the *in silico* predictive model for NK fold expansion for various cytokine cocktails. Multiple (*n* = 64) imputation maps are created based on primary and secondary stats activated by IL‐2, 12, 15, 18, 21 (Supplementary table 2). Applying a feature selection method (RRelief^27^) on the weighted average of NK cell fold expansion (Supplementary table 1b) on day 9, we selected only six imputation maps (maps 8, 14, 16, 28, 42 and 44) based on the results obtained in inhibition experiments in **(a, b)**. Based on these six imputation maps (Supplementary table 2), multiple linear regression models are constructed considering various interactions of activated STATs and NF‐κB between the priming and post‐priming I periods across different cytokine cocktail conditions that regulate NK cell fold expansion. To check the predictive ability of the models in estimating the weighted average of fold expansion on day 9 in response to different cytokine cocktails, leave‐one‐out cross‐validation (LOOCV)^26^ is performed by varying imputation maps and linear regression models (see the “Methods” section). The optimal imputation map and the regression model are chosen based on the minimum residual sum of squares (RSS) across 12 LOOCV test sets. **(e)** STAT and NF‐κB activation in the optimal imputation map (map 28) on day 9. **(f)** Comparison of the fold expansion between experiment (maroon) and *in silico* predictive model (gray) on day 9 for cytokine cocktail conditions 1‐12. The *in silico* regression model uses the optimal imputation map **(e)**. **(g)** Comparison of the predicted weighted average of fold expansion (**f**) with the experimental data across 12 cytokine cocktail conditions shows good agreement *(R* ^2^ = 0.55, see the “Methods” section for the calculation of *R* ^2^). **(h)** Predictions of the weighted averages of fold expansion on day 9 for conditions 2 and 3 with and without the presence of STAT3 and NF‐κB inhibitors using the optimal predictive model. **(i)** (left) Normalized distribution of the weighted average obtained from bootstrapped samples (*n* = 10000) for conditions 1–6 on an imputed fold expansion data on day 8. (Right) Means of the weighted averages with blue circles with 95% confidence interval represented by orange error bars. *P*‐values are calculated using the permutation test for each condition within the same group of donors (D1–D10) against condition 3. The red dashed line represents the average of all blue points across cytokine conditions 1–6.

To investigate the roles of Janus kinase/signal transducers and activators of transcription (JAK/STAT) and NF‐κB signaling pathways in IL‐21–mediated NK cell proliferation, NK cells were cultured using condition 2 (IL‐21 absent) or condition 3 (IL‐21 present) in the presence of either of STAT3 (S31‐201) or NF‐κB (PS1145) inhibitors and proliferation was measured. These results show that proliferation was dependent on both pathways for both conditions (Figure 3a, b). However, in condition 2, the effect of STAT3 inhibition was greater than that for NF‐κB (*P* = 0.0002), whereas the reverse was true for condition 3 (*P* = 0.0127) on day 9 (Figure 3a). Direct comparison of conditions 2 and 3 on individual donors confirmed that inhibition of the NF‐κB pathway had a more profound effect on condition 3 than on condition 2 (*P* < 0.0001), whereas the reverse was true for condition 2, where STAT3 inhibition had a higher effect than condition 3 (*P* = 0.0088; Figure 3b). To investigate the roles of phosphorylated forms of STAT3 (pSTAT3) and NF‐κB (pNF‐κB) in the early phase of NK expansion, we performed western blot experiments at early time points (1, 2, 5, 16, and 24 h) and compared the expressions of pSTAT3 and pNF‐κB for conditions 2 and 3 along with IL‐15 treatment. For all time points, in the absence of inhibitors, pSTAT3 was expressed higher in condition 3 than in condition 2 (Figure 3c). This suggests that IL‐21 exerts its additional pro‐proliferative effect predominantly via STAT3 in condition 3. Whereas we found similar levels of pNF‐κB expressions for conditions 2 and 3 which peaked at 2 h post‐priming (Figure 3c) and subsequently declined. This suggests that the early activation of pNF‐κB was regulated by IL‐18 present in the priming period for both conditions 2 and 3. We therefore presumed that the later effects of NF‐κB in fold expansion for condition 3 were related to transcription events caused by a synergy of IL‐21 signaling with NF‐κB activation. As a result of the complexity of cytokine signaling in NK cells, we undertook a modeling approach to further interrogate how they may synergize to affect NK cell proliferation.

### Interactions between STATs and NF‐κB induced by priming and post‐priming cytokine stimulation regulate NK cell proliferation *in vitro*

Cytokine stimulation induces NK cell modulation by signaling via transcription factors such as STATs and NF‐κB. Experiments have identified how specific cytokines are related to these STATs or NF‐κB (Table 1). However, many interactions are possible as the same STATs and NF‐κB can be activated by multiple cytokines. In addition, there is crosstalk between the STAT and NF‐κB pathways, which induces NK cell proliferation, and this crosstalk is dependent on the cytokines used to stimulate the NK cells. We created various possible imputation maps for our *in silico* studies that explored potential hypotheses for primary and secondary STAT/NF‐κB induced by specific cytokines (ILs) (Supplementary table 2). For example, IL‐2 primarily activates STAT5, but can also activate STAT1, STAT3, STAT4 as secondary STATs and nuclear factor NF‐κB, whereas IL‐18 primarily activates NF‐κB but can also activate STAT3 and STAT1 as secondary STATs (Table 1). We considered the presence of activated forms of STATs and NF‐κB, which affect NK cell proliferation with binary variables (0 ≡ absent, 1 ≡ present). Subsequently, a feature selection procedure using an algorithm (RRelief^27^) was performed to determine a subset of imputation maps (imputation maps 8, 14, 16, 28, 42 and 44; Figure 3d and Supplementary table 2) that are consistent with the experimental observation that STAT3 and NF‐κB inhibition downregulates NK cell proliferation when cultured with both condition 2 and 3 (Figure 3a). Next, we set up linear regression models to determine the effect of the crosstalk (synergistic or antagonistic) between the STATs and NF‐κB activated by the cytokine stimulations during the priming and the PP‐I stages in NK cell proliferation (see the “Methods” section). We reasoned that activated STATs and NF‐κB modify the rate at which single NK cells proliferate. Thus, we set up regression models where the weighted average of fold expansion of the NK cell population at a specific time point (e.g. day 9) with respect to its value in the unstimulated condition on day 0 is determined by the presence and absence of the STAT and NF‐κB activation in the priming and post‐priming stages. The weighted average in fold expansion is obtained by calculating weights for individual donors based on their overall NK fold expansion in different cytokine conditions (further details of the weighted average are in the “Methods” section; Supplementary table 1b). We trained or estimated the regression hyperparameters for a set of cytokine conditions and then predicted the weighted average of fold expansion of NK cell population for a cytokine condition not included in the training (Figure 3f). For our regression model, we considered four possible scenarios of STAT–NF‐κB interactions that regulate the fold expansion of NK cell populations (see the “Methods” section for details; Figure 3d) as follows: (1) STATs and NF‐κB induced in the *priming phase* but not in the post‐priming (PP‐I) phase regulate NK cell proliferation; (2) STATs and NF‐κB induced in the *PP‐I phase* but not in the priming phase regulate NK cell proliferation; (3) STATs and NF‐κB induced in the *priming and post‐priming (PP‐I)* regulate NK cell proliferation *additively*; (4) STATs and NF‐κB induced in the *priming and post‐priming (PP‐I) synergize or antagonize* and regulate NK cell proliferation (further details in the “Methods” section; Supplementary figure 1 and Supplementary table 3). We fitted the regression models with LASSO regularization (L1)^26^ to the NK cell fold expansion data for each of the aforementioned scenarios. The NK cell fold expansion data show large variation across the donors, with outliers (super donors) exhibiting very high NK cell expansion for a particular cytokine cocktail compared with other donors (see donor 7 for condition 3 in Supplementary table 1a). Thus, we computed a weighted average of the fold expansion based on the variation in response of donors across different cytokine conditions in the NK cell populations by using deviances (details in the “Methods” section and Supplementary table 1b). The regression models for various scenarios and feasible maps are compared based on their prediction capabilities in terms of cross‐validation error,^26^ where, to predict the fold expansion for each observed cytokine cocktail condition or test data, we trained the regression model with the other observed 11 cytokine cocktail conditions in Figure 1c and predicted the proliferation in the condition left out in the training by performing leave‐one‐out cross‐validation (LOOCV). This was performed for all possible choices (12 in total) where proliferation in each of the conditions was predicted. Finally, to determine the optimal imputation map and regression model, we evaluated their performance using a standard measure of overall prediction error: the residual sum of squares (RSS).^26^ This evaluation was conducted across 12 LOOCV test sets, with each set representing a different cytokine cocktail condition. For each regression model, *R* ^2^ (see the “Methods” section) is calculated to evaluate overall predictions for those 12 observed conditions (Supplementary table 3, Supplementary figure 1). Our best predictive model selects imputation map 28: IL‐2 → STAT5, STAT4, STAT1, IL‐12 → STAT4, IL‐15 → STAT5, IL‐18 → NF‐κB, STAT3, IL‐21 → STAT3, STAT1 (Figure 3e) among those selected feasible imputation maps with a strong pairwise synergy between STAT3 in the priming and STAT1 in post‐priming (PP‐I) stimulation phases (*linear‐Priming* ⊗ *linear‐PP‐I* in the “Methods” section and Supplementary table 4) is best suited (with the minimum RSS and the highest *R* ^2^; see the “Methods” section, Supplementary table 3 and Supplementary figure 1) to describe the fold expansion in NK cell populations.^26^ Using the optimized regression model, we predicted the fold expansion for each condition and compared these predictions with the observed data in Figure 3f. For the prediction against each *i*th (*i* = 1, …, 12) cytokine condition, we trained the model using the other observed 11 conditions *j* (*j ≠ i*) in experiments (Figure 3f) and then predicted the fold expansion for the excluded condition. Our predictive model performs reasonably well (*R* ^2^ > 0.5) to predict the fold expansion for most of the cytokine conditions. The model predicts that cytokine condition 3 generates the maximum fold expansion for typical donors compared to the other observed conditions that are validated in the experimental data (Figures 1c and 3f). To verify the statistical significance of fold expansion in condition 3 compared to other cytokine conditions, we imputed the data for day 8 among the same group of donors (*n* = 10) and calculated the weighted average for each condition. The weight assigned to each donor was based on NK cell viability at early days (days 2 and 4) and variability on day 8 to reduce the influence of outliers (see the “Methods” section). We compared the weighted averages across all conditions 1–6 using Fisher's least significant difference test,^29^ which revealed that the weighted averages of fold expansion in conditions 1–6 are not equal (*P* = 0.005) (Figure 3i, left). We further validated this finding by employing a permutation test,^30^ which showed that the weighted average for condition 3 is significantly higher than the other conditions: condition 1 (*P* = 0.03), condition 2 (*P* = 0.02), condition 4 (*P* = 0.03), condition 5 (*P* = 0.01) and condition 6 (*P* = 0.04; Figure 3i, right) within the same group of donors. These analyses suggest that condition 3 exhibits a promising potential for greater expansion among conditions 1–6 within the same group of donors.

**Table 1:** List of activated STATs and NF‐κB by ILs.

Cytokines	Induced STATs/NF‐κB reported in the literature	References	Induced STATs/NF‐κB used in our model	Comments
IL‐2	**STAT5**, STAT3, STAT4, STAT1, NF‐κB	6, 31, 32, 33	**STAT5**, STAT3, STAT1, STAT4, NF‐κB	
IL‐12	**STAT4**, STAT3, STAT1, STAT5	6, 31	**STAT4**	For simplicity, we considered the primary STAT contribution alone
IL‐15	**STAT5**, STAT3, STAT1	6, 31, 34	**STAT5**	For simplicity, we considered the primary STAT contribution alone
IL‐18	**NF‐κB**, STAT3	35, 36	**NF‐κB**, STAT3	
IL‐21	**STAT3**, STAT1, STAT5	6, 31	**STAT3**, STAT1	Considered the primary and one secondary STAT contributions

The bold face and normal fonts represent the primary and the secondary STAT/NF‐κB transcription factors, respectively.

IL, interleukin; NF‐κB, nuclear factor kappa B; STAT, signal transducers and activators of transcription.

Next, we examined the predictive performance of the optimal model in determining the condition associated with the lowest fold expansion in the weighted average among the 12 conditions. Our model identified condition 7 as the lowest expanding condition, which was also validated by the experimental data (Figure 3f and Supplementary table 1b). We also used our model to predict the fold change in NK cell expansion for a new condition (condition 13) which contains IL‐12 + 15 + 18 + 21 in the priming period (0–16 h) and IL‐2 + 21 in the PP‐I period (16 h to 3 days) by training the model with conditions 1–12, which predicts that the presence of IL‐21 in the post‐priming‐ period in condition 13 would not be beneficial for fold expansion (see the “Methods” section and Supplementary figure 7).

In addition, using the optimal map 28 and regression model (*linear‐Priming* ⊗ *linear‐PP‐I*), we predicted the weighted averages of NK cell fold expansion on day 9 for conditions 2 and 3 with and without the presence of STAT3 and NF‐κB inhibitors (Figure 3h). Both the STAT3 inhibitor (S3I) and the NF‐κB inhibitor reduce fold expansion in condition 2, in agreement with the *in vitro* data (Figure 3b). Furthermore, the STAT3 inhibitor leads to a greater decrease in the fold expansion than the NF‐κB inhibitor for condition 2, reproducing the fold expansion behavior observed in experiments (Figure 3b). For condition 3, the STAT3 inhibitor in the model shows a reduced fold expansion, in agreement with experimental *in vitro* observations (Figure 3a). However, the reduction in the fold change in condition 3 was weaker for the NF‐κB inhibitor and had a lesser effect than the STAT3 inhibitor in reducing fold expansion, indicating a limitation in the training of our model.

To improve the accuracy of predictions, more experimental cytokine conditions should be included in the training of the *in silico* model. At present, our model considers the activation of STATs and NF‐κB in binary representation using imputation maps. The measurements of concentrations of activated STATs and NF‐κB in future experiments will help incorporate their dependence, which could differ between the cytokine conditions and provide a better quantitative agreement of model predictions with the data. Despite these limitations, our model provides reasonably good predictions (*R* ^2^ > 0.5) for most conditions and valuable quantitative insights into the contributions of STAT3, NF‐κB and other induced STATs during the priming and post‐priming phases. It also sheds light on the intricate interplay and potential synergistic or antagonistic interactions among these factors in driving the proliferation of NK cells under the influence of different cytokine cocktails (Supplementary table 4). Overall, our model suggests that condition 3 is a high expanding culture condition among the observed 12 conditions. This indicates that IL‐21 induces enhanced NK cell expansion under the experimental conditions tested. Conversely, condition 7, lacking IL‐21 in the priming phase and IL‐15 in the post‐priming phase, exhibits the lowest weighted average of NK cell fold expansion  (Figure 3f and Supplementary table 1b).

### The effect of cytokines on NK cell function

**Figure 4:**
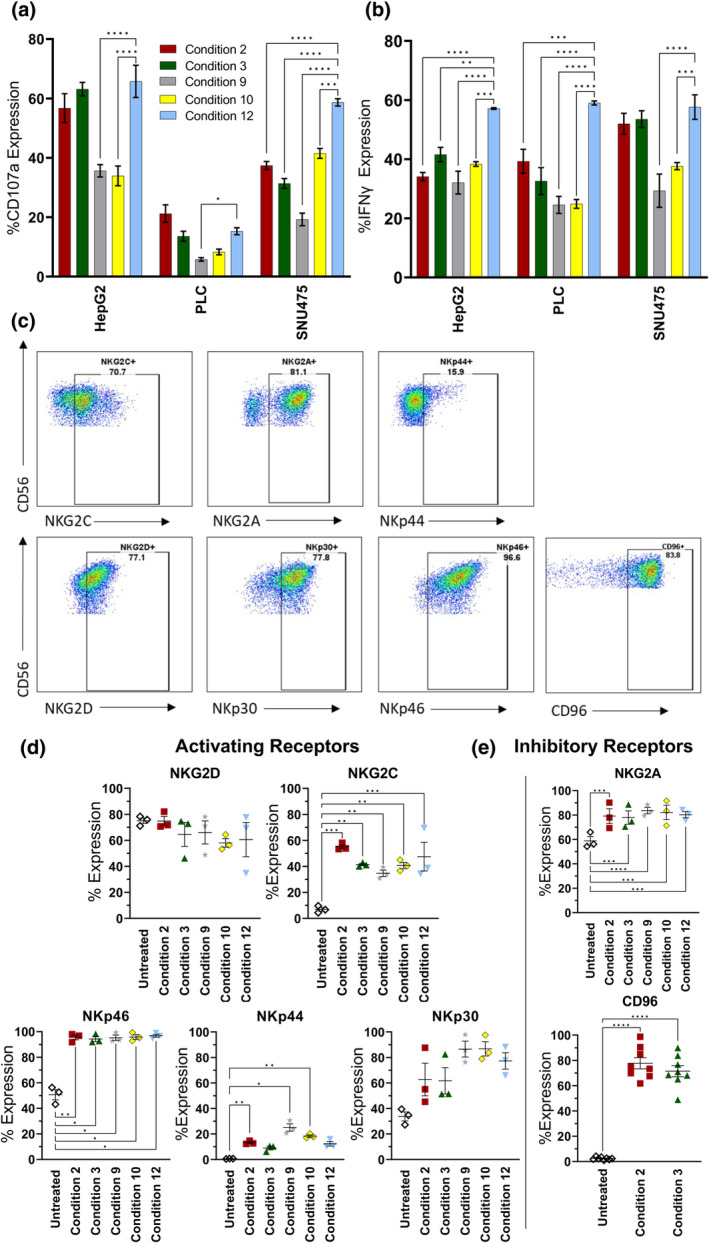
Effects of different regimens on natural killer (NK) cell receptor expression and cytotoxicity potential against hepatocellular carcinoma cell lines as a model for immunotherapy. NK cells from three donors (D15–D17) were cultured using the indicated cytokine conditions and then assayed on day 10 for cytotoxicity potential against the indicated hepatocellular carcinoma cell lines using a CD107a degranulation assay **(a)** or for expression of interferon gamma (IFNγ) **(b)**. Assays were performed at an effector‐to‐target ratio of 1:1. Data were analyzed by two‐way ANOVA and Dunnett's multiple comparison test (*P ≤ 0.05, **P ≤ 0.01, ***P ≤ 0.001, ****P ≤ 0.0001). **(c)** Representative example of gating strategy. CD56^+^ NK cells were analyzed for the expression of NKG2D, NKG2C, NKp46, NKp44, NKp30, NKG2A and CD96 by flow cytometry. **(d)** Expression of activating receptors by NK cells stimulated by cytokines for 10 days. **(e)** Expression of inhibitory receptors by NK cells stimulated by cytokines for 10 days. Data are shown as mean ± standard error of the mean (*n* = 3), analyzed by Repeated Measures (RM) one‐way ANOVA and Dunnett's multiple comparison test (***P ≤ 0.001, ****P ≤ 0.0001). Untreated indicates freshly isolated NK cells without cytokine addition. These were analyzed by flow cytometry directly after isolation and were not kept in culture with cytokines. On day 10 of culture, cytokine‐stimulated NK cells were analyzed by flow cytometry, and their phenotype was compared with the untreated NK cells.

Successful immunotherapy depends on NK cell functionality as well as NK cell numbers. Therefore, to determine how the different cytokine combinations affected NK cell effector functions, we studied degranulation (CD107a) and IFNγ production using NK cells cultured under different protocols with and without IL‐21 (Figure 4a, b). These were then tested in coculture assays with three different hepatocellular carcinoma (HCC) cell lines on day 10 of culture. The introduction of IL‐21 in the priming phase did not affect the cytotoxicity potential or IFNγ secretion as determined by a comparison of condition 2 with condition 3. The overall effector function was most notable for condition 12, which gave the strongest results for both degranulation and IFNγ secretion against the cell lines tested. This regimen contains IL‐21 in the priming phase and IL‐18 in the post‐priming phase, and strong cytotoxicity was seen against two out of the three cell lines tested, with high levels of IFNγ secretion against all the cell lines tested (Figure 4a, b). Thus, it appears that the dominant effect on cytotoxicity potential occurred when IL‐18 was present in the post‐priming phase.

To correlate the effector functions with cytokine conditions, we studied receptor expression. Overall, there were no simple correlations of receptor expressions with cytokine condition. In particular, the expression of the inhibitory receptor NKG2A was high for all regimens tested and the expression of the activating receptors NKG2D, NKG2C and NKp46 was similar among the different regimens (Figure 4d, e). There was a trend toward lower NKp44 and NKp30 in the conditions associated with IL‐12/15/18 priming.

**Figure 5:**
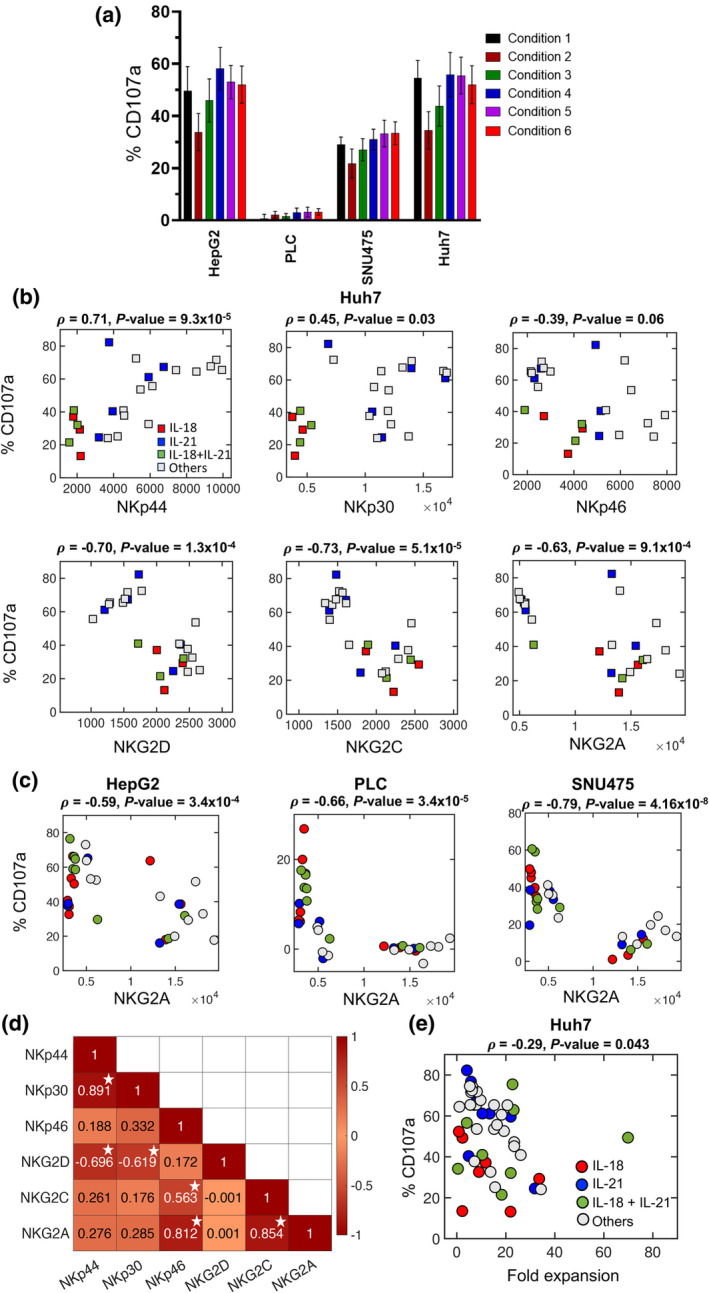
Correlations of cytotoxicity potential with natural killer (NK) cell proliferation and receptor expressions against different hepatocellular carcinoma cell lines. **(a)** %CD107a expression levels against four hepatocellular carcinoma cell lines HepG2, PLC, SNU475 and Huh7. NK cells from eight donors (D3–D10) were incubated with cytokines according to conditions 1–6 and then assayed for proliferation, CD107a and receptor expression on day 10. **(b)** Correlation of cytotoxicity potential (%CD107a expression) against the Huh7 cell line with mean NK cell receptor expressions for the high CD16‐expressing donors (8 donors: D5 and D6, D8–D10, D15–D17) cultured under 10 different cytokine conditions (conditions 1–6, 9–12). Each square represents a donor, labeled based on the treatment conditions in the presence of either interleukin (IL)‐18 (red) or IL‐21 (blue) or IL‐18 + 21 (green) or none of the two (gray). Pearson correlations (*ρ*) between %CD107a and each of mean receptor expressions are shown along with *P‐*values. **(c)** Correlation of NKG2A expression on NK cells with cytotoxicity potential for HepG2, PLC and SNU475 cell lines showing Pearson correlations (*ρ*) and *P*‐values from high CD16‐expressing donors. **(d)** Correlation matrix representing the correlation between the mean receptor expressions on the NK cells from high CD16‐expressing donors. Pearson correlation coefficient values are shown in each box. A darker color indicates a higher correlation (positive or negative) between the two receptor types. Boxes with white stars indicate Pearson correlation values with *P* < 0.05. **(e)** Against the Huh7 cell line, %CD107a expression from **(a)** was correlated with the average of NK cell fold expansion on day 9 (Figure 1c) for eight donors (D3–D10), each treated with conditions 1–6.

To further compare the cytotoxic responses of NK cells against HCC cell lines, we used a different set of donors and measured CD107a expressions against HepG2, PLC, SNU475 and Huh7 using a different combination of cytokines (conditions 1–6). This allowed assessment of the use of IL‐21 in the priming (condition 3) and post‐priming periods (condition 6) (Figure 5a). We observed the highest cytotoxic responses against HepG2 and Huh7 cells, with a moderate response against SNU475 and a minimal response against PLC. To investigate how receptor expression determined the cytotoxicity potential of NK cells in response to these cell lines, we performed correlation analysis to evaluate Pearson correlation coefficients between expressions of CD107a and activating/inhibitory receptors (Supplementary figures 2–4 and Supplementary table 5). However, it did not generate any positive correlations of cytotoxicity potential with the expression of any specific activating receptor for these cell lines except Huh7 (Figure 5b). We found that CD107a expression on CD56^+^CD16^+^ cytotoxic NK cells had a positive correlation with levels of the activating receptor NKp44 (*ρ* = 0.71, *P* = 9.3 × 10^−5^) for the Huh7 cell line but there were no other positive correlations noted with the other receptors (Figure 5b). Conversely, there was a negative correlation with the inhibitory receptor NKG2A (*ρ* = −0.63, *P* = 9.1 × 10^−4^) (Figure 5b). This is extended to the other cell lines (HepG2, PLC and SNU475; Figure 5c). Thus, using multiple NK cell expansion protocols, the dominant determinant in killing HCC cell lines appears to be NKG2A expression. We further observed that within these NK cell expansion protocols, the activating receptor NKG2C, expressed in adaptive and memory‐like NK cells, correlated positively with the inhibitory receptor NKG2A (*ρ* = 0.854, *P* = 2.8 × 10^−10^). In addition, there were positive correlations between the following pairs of activating receptors: NKp30 and NKp44 (Pearson correlation, *ρ* = 0.891, *P* = 3.5 × 10^−12^) and NKG2C and NKp46 (*ρ* = 0.563, *P* = 6.5 × 10^−4^), and negative correlations between NKG2D and NKp30 (*ρ* = −0.619, *P* = 1.2 × 10^−4^) and NKG2D and NKp44 (*ρ* = −0.696, *P* = 6.9 × 10^−6^; Figure 5d).

Finally, we determined how proliferation impacted NK cell cytotoxicity potential. For the Huh7 cell line, using the six different regimens (conditions 1–6) tested in eight donors (D3–D10), we observed a generally negative impact of proliferation on cytotoxicity potential (*ρ* = −0.29, *P* = 0.043), with one donor showing both strong proliferation and cytotoxicity (Figure 5e, donor 7 for condition 3 in Supplementary table 1a). However, there was no clear segregation of regimens using the different cytokine combinations (other cell lines in Supplementary figures 2–4).

**Figure 6:**
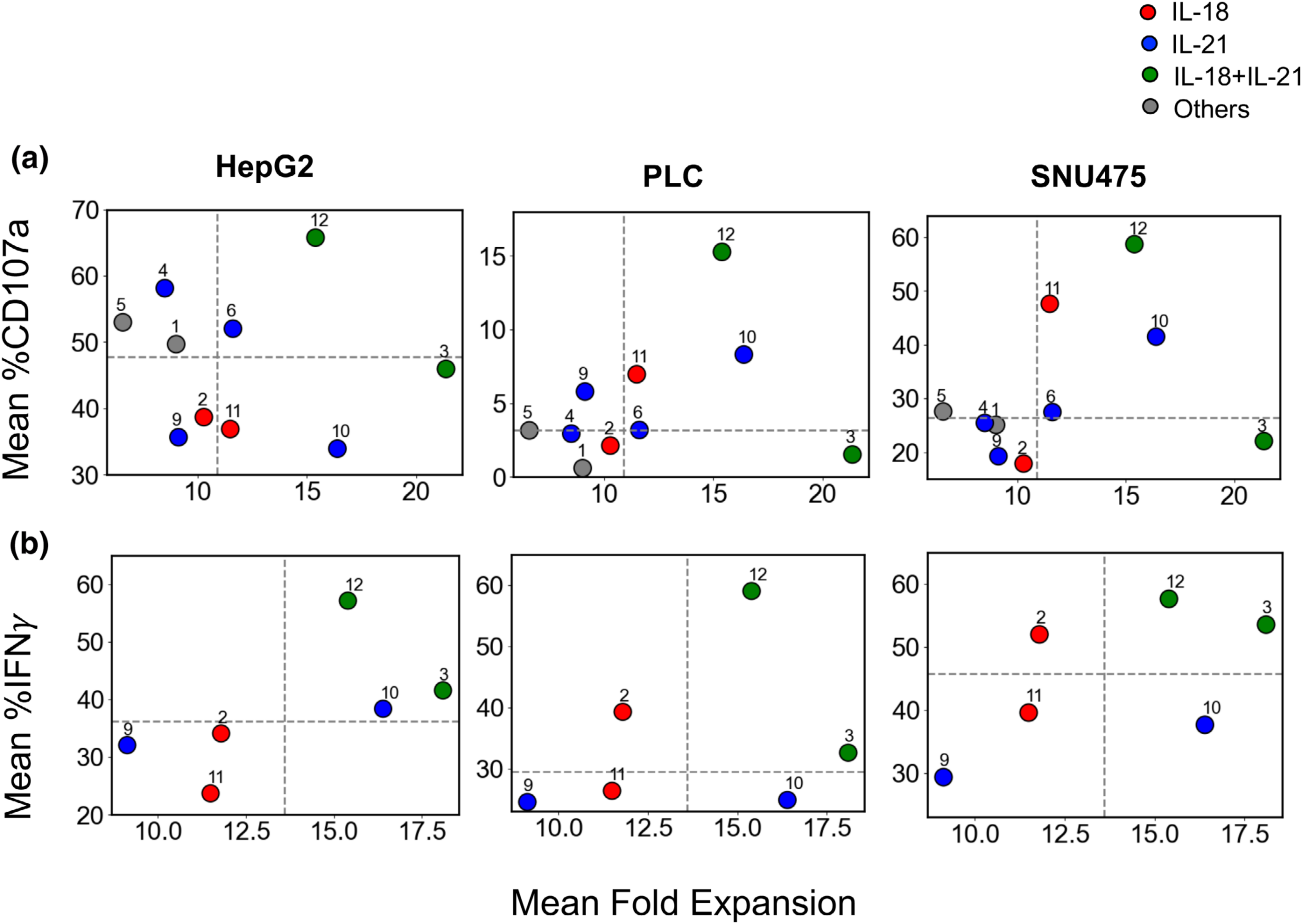
Covariation of the mean of percentage degranulation and interferon gamma (IFNγ) secretion against different hepatocellular carcinoma cell lines with mean fold expansion of proliferating natural killer (NK) cells under different cytokine treatment conditions. **(a)** Variation of mean %CD107a expressions in NK cells responding to HepG2, PLC and SNU475 cell lines on day 10 is shown against the mean fold expansion of NK proliferation on day 9 under different cytokine treatment conditions. %CD107a and %IFNγ expressions are measured in expanded NK cells that were cocultured with hepatocellular carcinoma cell lines. The fold expansions of NK cells were measured under different cytokine treatment conditions as described in the main text. Each circle represents the mean values calculated for each cytokine treatment condition, with the cytokine conditions denoted by numbers above the circles. For conditions 1–6, the averages are calculated using eight donors (D3–D10) and for conditions 9–12, the averages are calculated using three donors (D15–D17). Colors filling the symbols depict the presence of interleukin (IL)‐18 (red) or IL‐21 (blue) or both (green) or none of those (gray) in the cytokine treatment condition. The dashed lines represent the medians of the respective data. **(b)** Variation of mean %IFNγ expressions on day 10 in NK cells responding to HepG2, PLC and SNU475 cell lines with mean fold expansion of NK proliferation on day 9 under different cytokine treatment conditions. Each circle represents the mean values calculated for each cytokine treatment condition using three donors (D15–D17).

For successful immunotherapy applications, NK cell expansion, degranulation and cytokine production are crucial. However, the greatest expanding NK cell subpopulation generally consists of immature cells with reduced degranulation capacity. By contrast, more mature NK cells show better degranulation, but exhibit decreased expansion rate. Thus, to determine the trade‐off between NK cell proliferation and degranulation or cytokine release in our culture conditions, we plotted average proliferation on day 9 as a function of the average %CD107a and %IFNγ expressions across HCC cell lines HepG2, PLC and SNU475 on day 10, which was the closest to the observed day of the proliferation measurement. We observed that across all cell lines, condition 12 yielded a significant mean proliferation, along with notable mean percentages of CD107a and IFNγ expressions (Figure 6a, b). By contrast, condition 3 showed higher NK cell expansion, with elevated IFNγ expressions across all cell lines and moderate degranulation in HepG2, but limited degranulation against PLC and SNU475. Overall, these observations suggest that cytokine conditions 12 and 3, which include IL‐21 in the priming and IL‐18 in the post‐priming‐ phase, could be beneficial for immunotherapy treatment and should be explored in more detail in future studies.

## DISCUSSION

Characterization and profiling of NK cells are essential to better understand their biology, so they can be modulated for future NK cell–based immunotherapy. There is a complex interplay between cytokines that dictates the functions of NK cells, but this needs to be understood in order to identify better combinations of cytokines for clinical use. We address this challenge by developing an *in silico* framework to predict fold expansion in populations of NK cells treated with cytokine cocktails. Our results indicate that NK cell proliferation can be modulated during both the short 16‐h priming and a 3‐day post‐priming period. Our modeling suggests the importance of synergy between STAT3, induced by IL‐21, in the initial priming period  with other STATs, such as STAT1, in the post‐priming period in increasing the NK cell proliferation (Supplementary table 4). Previous studies have demonstrated the roles of STAT3 in conjunction with STAT5 in suppressing NK cell cytotoxicity;^6^ however, our experimental and modeling studies demonstrate an additional synergistic role of STAT3 in increasing NK cell proliferation.

We focused primarily on IL‐21, as this has been used predominantly in a membrane‐bound form to induce the proliferation of NK cells, but we sought to identify opportunities for using this cytokine in its physiological soluble form to investigate its potential without the use of feeder cells. IL‐21 induces STAT3 activation leading to downstream activation of c‐Myc which regulates cellular processes such as increasing NK cell proliferation, induction of glycolysis, mitochondrial biogenesis and the cell cycle.^37^ NK cells with a high STAT3 expression following IL‐21 stimulation have rapid proliferation, long telomeres and resistance to senescence.^37^ This may be important in making NK cells more resistant to immunosuppressive tumor microenvironments and hence enhance their activity against “cold” tumors, such as HCC.^38^, ^39^, ^40^, ^41^ Solid tumors are less susceptible to NK cell therapies than hematological tumors, and therefore, making NK cells less susceptible to these immunosuppressive effects will be key to generating successful NK cell–based treatments for this class of cancers. Although IL‐21 primarily signals via a STAT3, NF‐κB inhibition caused a lower proliferation in the presence of IL‐21, indicative of the complex interplay between these two pathways on NK cell proliferation, which has previously been observed in cancer.^42^ Our *in silico* model points to a synergistic interaction between STAT3 activated in the priming and NF‐κB activated in PP‐I (the strength of the synergy of $S_{3}{\tilde{S}}_{b}$ is estimated in Supplementary table 4) in regulating NK cell expansion. Binding IL‐21 to its receptor activates STAT3. It is possible that STAT3 target genes then induce NF‐κB activation, leading to increased proliferation.^43^ In addition, given the potential for both IL‐21 and IL‐18 to upregulate NF‐κB, inhibiting this transcription factor may exert a more pronounced suppressive effect on proliferation. The importance of IL‐21 and NF‐κB in mediating NK cell expansion is highlighted by the significant reduction of NKG2A levels following NF‐κB inhibition, with expression decreasing from ~70% to ~35% in the presence of IL‐21 (condition 3, Supplementary figure 6b). This is in line with Kaulfuss *et al*.,^44^ who showed that the expansion of NK cells was greater in NKG2A^+^ NK cells and that this was not dependent on the STAT3 signaling pathway. It is possible that proliferation of NKG2A+ NK cells might be favored to maintain self‐tolerance and protect against tissue damage; however, these cells present a dampened antitumor response. This is consistent with our data showing the trade‐off between proliferation and killing, and the negative effects of NKG2A expression on NK cells when tested against all HCC targets. Therefore, his warrants further investigations into the abrogation of NKG2A expression, for potential therapeutic practices, albeit balanced by the expression of other inhibitory receptors to ensure maintenance of self‐tolerance and avoid activation‐induced cell death of NK cells.

Our data show that IL‐21–induced NK cells have a phenotype canonical of both immature and mature NK cells, expressing CD16 and NKG2A, but lacking CD57. We propose a model where NK cell population comprises several subgroups with different phenotypes and functions, in line with Björkström *et al*.^45^ We hypothesize that NK cell population maturation occurs in waves, where older CD57^+^ senescent NK cells die off and the highly prolific CD57^−^ cells take over and form the largest part of the population. This indicates that several phenotypical combinations can be present at any one time within the NK cell pool.

A major challenge in predicting changes in population sizes of NK cells treated with cytokine cocktails is the differences in the induction of phosphorylated forms of STAT and NF‐κB factors which can potentially involve a large number (~100) of combinations of STAT and NF‐κB factors that can synergize/antagonize to affecting NK cell proliferation. In the cytosol, the STATs form homo‐ and hetero‐dimers, and in certain cases tetramers, where the formation of specific multimers can depend on the concentrations of phosphorylated forms of STATs generated during signaling.^46^, ^47^, ^48^ The STAT dimers can further synergize/antagonize in the nucleus where these might competitively/co‐operatively engage with DNA regulatory elements and affect co‐operative gene regulatory processes.^13^, ^49^, ^50^ We confronted this challenge in large dimensions by implementing a feature selection algorithm RRelief for fold expansion of NK cell populations in treatment conditions containing different combinations of ILs and repeating some of these experiments with STAT3 and NF‐κB inhibitors. The combined approach allowed us to select a small number of potential possibilities for STAT and NF‐κB induction in the cytokine cocktails that are consistent with the inhibition experiments (Figure 3a). The next challenge of relating the phosphorylation of STAT and NF‐κB factors, their synergy/antagonism and their influence on proliferation was addressed by using a linear regression model with LASSO regularization. Cross‐validation of the possible models against the NK cell fold expansion data revealed that the synergy/antagonism between the transcription factors induced in the priming and post‐priming play an important role in affecting NK cell proliferation. The success of our framework in predicting the cytokine cocktail with the largest fold expansion demonstrates the utility of such combined approach in modeling NK cell response against cytokine cocktails. This methodology, initially studied on day 9, holds promise for further investigation on other days of proliferation, and perhaps for modeling the maturation of NK cells in cytokine cocktails. Our work integrates NK cell proliferation, function and phenotype to develop a model for NK cell generation that supports the incorporation of IL‐21 into short‐term NK cell proliferation regimens. The next step would be to incorporate these into preclinical models for cancer immunotherapy.

### Limitations of the study

The main limitation of the biological aspects of this study is the use of multiple different donors, and not all were tested with the same regimens. This was a result of sample volume limitations; purified NK cells were used for these assays. However, this limitation may also be considered a strength of the work, as it is important to sample different donors to generate a model that is widely applicable to the population. This is because it is known that there is substantial donor‐to‐donor variability, which affects NK cell proliferation. It should also be noted that there was a substantial donor‐to‐donor variability which is a known feature of NK cell proliferation. Our regression models did not consider dependencies of NK cell proliferation on cytokine concentrations and subcellular concentrations of the induced STAT and NF‐κB proteins which could further regulate synergy/antagonism between these transcription factors. In this study, our model framework was employed to predict NK cell proliferation at day 9, which can be extended to study other days. However, the optimal map and regression model could be different at other days than day 9. For our optimal predictive model selection, the imputation map and regression model representing STAT–NF‐κB interactions chosen in our study might not uncover the true mechanism, yet serve as a crucial mathematical framework for generating inferences in the context of cytokine conditions.

## METHODS

### Peripheral blood mononuclear cell isolation and NK cell isolation

Peripheral blood mononuclear cells were isolated from buffy coat of healthy donors by density gradient centrifugation, using Ficoll–Hypaque (Fisher Scientific, Loughborough, UK) following the manufacturer's instructions. CD56^+^CD3^−^ NK cells were purified from peripheral blood mononuclear cells by positive selection using the Miltenyi NK isolation kit with LS columns (Miltenyi Biotec, Surrey, UK), as stated by the manufacturer's protocol.

### Cell lines

Liver cancer cell lines HepG2 and PLC were cultured in complete Dulbecco's modified Eagle medium (DMEM; Life Technologies, Paisley, UK) supplemented with 10% fetal bovine albumin (Sigma‐Aldrich, Gillingham, UK) and 5% PenStrep (Life Technologies, Paisley, UK). The liver cancer cell line SNU475 was cultured in complete Roswell Park Memorial Institute (RPMI; Life Technologies, Paisley, UK) medium, supplemented with 10% fetal bovine albumin and 5% PenStrep. All cells were maintained in a humidified incubator at 37°C with 5% CO_2_.

### NK cells stimulation

Natural killer cells were maintained in culture in NK MACS medium (Miltenyi Biotec, Surrey, UK), supplemented with 1% NK supplements (Miltenyi Biotec), 1% PenStrep (Life technologies, UK) and 5% human AB serum (Sigma‐Aldrich, Gillingham, UK). NK cells were expanded with cytokines IL‐2 (500 U mL^−1^), IL‐12 (10 ng mL^−1^), IL‐15 (20 ng mL^−1^), IL‐18 (50 ng mL^−1^) and IL‐21 (25 ng mL^−1^; all from R&D System, Abington, UK) for 10 days.

### NK cells staining

Natural killer cells were analyzed for receptor expression using the following antibodies: CD56 (BV510, BioLegend, London, UK), CD3 (APC/Cy7, BioLegend, London, UK), NKp30 (PerCp/Cy5.5, BioLegend), NKG2A (FITC, Miltenyi Biotec), NKG2C (PE, Miltenyi Biotec), NKG2D (FITC, BioLegend), CD94 (BV421, BioLegend), NKp44 (PerCp/Cy5.5, BioLegend), NKp46 (APC, BioLegend), CD16 (BV421, BioLegend), CD57 (APC, BioLegend), CD117 (PE/Cy7, BioLegend) and CD16 (BV421, BioLegend).

For carboxyfluorescein succinimidyl ester staining, unstimulated NK cells were stained with CellTrace carboxyfluorescein succinimidyl ester (Thermo Fisher Scientific, Paisley, UK), according to the manufacturer's instructions and assessed for staining by flow cytometry. Data were analyzed using FlowJo version 10.9.0.

### Degranulation assay and assessment of IFNγ expression

Natural killer cells were cultured with the cell line in complete RPMI medium, along with anti‐human CD107a‐eFluor660 (clone H4A3, Invitrogen, Paisley, UK), at an E:T ratio of 1:1 for 4 h at 37°C. After 1 h of incubation, GolgiStop Protein Transport Inhibitor (BD Bioscience, Wokingham, UK) was added, and the cells were incubated for an additional 3 h. Cells were then stained with antibodies CD56 (PE/Cy7, BioLegend), CD3 (BV510, BioLegend) and IFNγ (PE, BioLegend) and analyzed by flow cytometry. The gating strategy is shown in Supplementary figure 5.

### Statistics

Data were analysed with GraphPad Prism 9, using one‐way ANOVA or two‐way ANOVA where appropriate, as specified in the figure captions. Significance values are indicated as *P ≤ 0.05, **P ≤ 0.01, ***P ≤ 0.001, ****P ≤ 0.0001.

### Model development

#### Terminology for priming and post‐priming (I and II)

The experimental procedure consists of three distinct stages: priming, PP‐I and PP‐II (Figure 1b). During the priming stage, NK cells were exposed to specific cytokine cocktails for 16 hours. This initial treatment was designed to activate and prepare the NK cells for subsequent analysis. After the priming stage, the culture media was replaced, and the cells are subjected to a new set of cytokine cocktails. This second phase, lasting from 16 hours to 3 days, is referred to as the PP‐I period (Figure 1b). Media was again replaced on day 3 and a new set of cytokines were added for days 3 to 16, which is referred to as the PP‐II period.

#### Calculation of the weighted average (Y) for NK cell fold expansion

The NK cell proliferation data show high variability in fold expansion across donors. Thus, to choose a better representative quantity, instead of calculating the standard average of fold expansions across donors (Figure 1c), we calculated the weighted average for the fold expansion (Figure 3f and Supplementary table 1b). Here, we considered unequal weights for individual donors based on their responses in fold expansion across various cytokine cocktail conditions. For example, if NK cells from a donor are treated with *k* cytokine cocktail conditions (e.g. conditions 1–6, i.e. *k* = 6), we calculated the variances $\left(\sigma_{i}^{2}=\mathrm{var}\left(\left\{f_{i}^{1},f_{i}^{2},\ldots ,f_{i}^{k}\right\}\right)\right)$ of NK cell fold expansion ($f_{i}^{a}$) across *α* = 1,…, *k* cytokine conditions for that *i*th donor. The data show that different donors have small, moderate or high variances in fold expansion across conditions. We assigned significant weights for donors with moderate variances in fold expansion across cytokine cocktail conditions and less for donors with very high or low variances. In such a way, we assigned small weights to the outliers either to non‐responders (low variance in NK cell fold expansion across *k* cytokine conditions) or to super‐responders (very high variance in NK cell fold expansion across *k* cytokine conditions), and assigned higher weights to the typical responders displaying variances around the median of variances $\left(\mathrm{median}\left(\left\{\sigma_{1}^{2},\sigma_{2}^{2},\ldots ,\sigma_{N}^{2}\right\}\right)\right)$ in fold expansion from *N* donors. This is systematically done by choosing the weights for an individual donor (*ω*_i_) as inversely proportional to their deviance ∝ $\frac{1}{\text{devianc}e_{i}}$. By normalizing the weights, we get the weight for the *i*th donor as $\omega_{i}=\frac{\frac{1}{{\text{deviance}}_{i}}}{\sum_{i=1,\ldots ,N}\;\left(\frac{1}{\text{devianc}e_{i}}\right)}$, such that $\sum_{i=1,\ldots ,N}\omega_{i}=1$. Here, the deviance of the *i*th donor $d_{i}={\left(\sigma_{i}^{2}-\text{medme}\right)}^{2}$, where *medme* = [0.1 × *mean* $\left(\left\{\sigma_{1}^{2},\sigma_{2}^{2},\ldots ,\sigma_{N}^{2}\right\}\right)$ + 0.9 × *median* $\left(\left\{\sigma_{1}^{2},\sigma_{2}^{2},\ldots ,\sigma_{N}^{2}\right\}\right)]$. The weighted average of fold expansion for *α*th cytokine condition is calculated as $Y^{\alpha }=\sum_{i=1}^{N}\omega_{i}{f_{i}}^{a}$, where $f_{i}^{\alpha }$ represents the fold expansion for the *i*th donor treated with *α*th cytokine condition.

In our data (Supplementary table 1a), because all 17 donors are not treated with the same cytokine conditions, we divided them into three groups, namely, (1) Group A: *N*_*A*_ (=10) donors are treated with conditions 1–6 (donors 1–10), (2) group B: *N*_*B*_ (=4) donors are treated with conditions 7 and 8 (donors 11–14) and (3) group C: *N*_*C*_ (=3) donors are treated with conditions 9–12 (donors 15–17), and calculated the weighted average of fold expansion $Y_{A}^{\alpha_{A}}=\sum_{i=1}^{N_{A}}\omega_{i}{f_{i}}^{a_{A}}$, $Y_{B}^{\alpha_{B}}=\sum_{i=1}^{N_{B}}\omega_{i}{f_{i}}^{a_{B}}$ and $Y_{C}^{\alpha_{C}}=\sum_{i=1}^{N_{C}}\omega_{i}{f_{i}}^{a_{C}}$ for the *α*th cytokine condition for groups A, B and C, respectively. We then combined them to calculate the weighted average of the fold expansion $Y=\left\{Y_{A},Y_{B},Y_{C}\right\}$ across 12 conditions.

#### Generation of imputation maps: IL to STAT/NF‐κB conversion

Cytokines might induce synergistic or antagonistic effects on NK cell responses (proliferation or degranulation). Each cytokine activates multiple STATs, which include a primary STAT along with secondary STATs (Table 1). As the type of activated STATs by a particular cytokine is limited (seven), the STATs and NF‐κB induced by different cytokines can overlap. Consequently, different cytokine cocktails can activate the same sets of STATs, albeit in different concentrations. IL‐2 and IL‐15 activate primarily STAT5, whereas IL‐12, IL‐18 and IL‐21 primarily activate STAT4, NF‐κB and STAT3, respectively. In addition, IL‐2 activates STAT1, STAT3, STAT4 and NF‐κB as secondary STATs. By contrast, IL‐18 and IL‐21 activate STAT3 and STAT1, respectively, as secondary STATs.

In our modeling framework, we considered the presence or the absence of STATs in a binary representation (0 or 1) because of the lack of knowledge about the true concentrations of activated STATs for a specific cytokine cocktail. In addition, it is unclear what set of secondary STATs is activated by a particular cytokine cocktail. Therefore, we assumed in our model that each IL in a cytokine cocktail activates either a primary STAT alone or a primary STAT along with a set of secondary STATs and NF‐κB chosen from all possible STAT and NF‐κB combinations (single or multiple; Table 1). For example, IL‐2 can induce STAT5 as a primary STAT alone or STAT3, STAT4, STAT1 and NF‐κB as secondary STAT/NF‐κB along with it in 16 (=1 + 4C_1_ + ^4^C_2_ + ^4^C_3_ + ^4^C_4_) ways. Similarly, IL‐12, IL‐15, IL‐18 and IL‐21 can induce secondary STATs and NF‐κB in 1, 1, 2 and 2 ways, respectively. Considering all the possible combinations of activated STATs and NF‐κB in a cytokine cocktail, there can be up to 16 × 1 × 1 × 2 × 2 = 64 possible sets (indexed by *i*) of primary and secondary STATs (Figure 3d and Supplementary table 2). Each of these possibilities represents a specific imputation map (indexed by *i*) which generates activated STATs and NF‐κB as the outputs for the cytokine combinations used in the treatment conditions. We created a binary matrix, **M**_*i*_ (*p,q*) of size 12 × 10, where the rows (*p*) and the columns (*q*) represent the cytokine conditions and the STATs and NF‐κB, respectively, and the matrix elements describe the presence or the absence of STATs and NF‐κB for any cytokine condition given by the imputation map *i*. Columns 1–5 and 6–10 represent the presence or absence of STAT1, STAT3, STAT4, STAT5 and NF‐κB during priming and PP‐I, respectively. Thus, by applying these possible 64 imputation maps to cytokine treatment conditions, we constructed 64 binary matrices **M**_*i*_, where, *i* = 1, …, 64.

#### Selection of imputation maps based on inhibition experiments

We selected imputation maps that can describe the observations with STAT3 and NF‐κB inhibitors in the following way. We imposed two constraints observed in the experiments with the inhibitors (see Figure 3a). *Constraint 1*: The STAT3 inhibitor in condition 2 significantly decreases the NK cell fold expansion. Thus, we imposed a constraint that condition 2 must induce STAT3. *Constraint 2*: STAT3 and NF‐κB inhibitors significantly decrease the NK cell fold expansion for conditions 2 and 3. Therefore, we imposed the constraint that the presence of STAT3 and NF‐κB increases NK cell proliferation. We evaluated the contribution of each STATs and NF‐κB during priming and PP‐I to NK cell fold expansion on day 9 in terms of the weights (or scores) in the range of [−1, +1] using a feature selection method RRelief.^27^ Using the weighted average of NK cell fold expansion data on day 9 (***Y***) (Supplementary table 1b), we first obtained the weight (or score) of each STATs/NF‐κB during priming and PP‐I for an imputation map *i* as a vector ***W***_***i***_. Imposing the aforesaid constraints, we selected those maps for which ***W***_***i***_ contains positive weights corresponding to STAT3 and NF‐κB both in priming and in PP‐I (Figure 3d) to ensure STAT3 and NF‐κB arise as essential feature variables in regulating NK cell proliferation in the feasible imputation maps. Using this filtering technique, we reduced the number of feasible imputation maps from 64 to 6 that are consistent with experimental observations (Figure 3d).

#### Construction of multiple linear‐regression models to predict the weighted average (Y) of NK cell fold expansion for a cytokine cocktail condition at a certain day

To train and predict the weighted average of fold expansions on day 9 under different treatment conditions, represented by a vector ***Y*** (Supplementary table 1b), we constructed multiple linear regression models ***Y*** = **X*β*** + ɛ,^26^ where **X** represents a binary matrix constructed considering the possible interactions between the STATs and NF‐κB in priming and PP‐I. In our notation, each element (indexed by α) of the ***Y*** vector represents a treatment condition. We considered linear or, linear and pair‐wise synergistic or antagonistic contributions of the induced STATs and NF‐κB in the priming and PP‐I in regulating NK cell fold expansion. The activation of STATs and NF‐κB are represented by binary vectors {***S***_***p***_} where a STAT or NF‐κB (indexed by *p*) is present (=1) or absent (=0) in the treatment condition given by the elements of the vector ***S***_***p***_. The contribution of the STATs and NF‐κB, represented by ***S***_***p***_^***(prime)***^ and ***S***_***p***_^***(PP‐I)***^, in the priming and PP‐I phase to the fold expansion on day 9 is quantified by C_α_^(prime)^ = ∑_p_ [*a*_p_[S_p_^(prime)^]_α_ + ∑_q,p ≠ q_ *b*_pq_[S_p_^(prime)^]_α_[S_q_^(prime)^]_α_] and C_α_^(PP‐I)^ = ∑_p_ [*c*_p_[S_p_^(PP‐I)^]_α_ + ∑_q,p ≠ q_ *d*_pq_[S_p_^(PP‐I)^]_α_[S_q_^(PP‐I)^]_α_], respectively. The coefficients *b*_pq_ and *d*_pq_ are set to zero if no synergy/antagonism is considered within the priming and post‐priming phases. We considered four scenarios (8 regression models, see Supplementary figure 1 and Supplementary table 4) for combining STAT and NF‐κB contributions in the priming and post‐priming phases to describe the weighted average of NK cell fold expansion.

1. ***Priming alone***: In this model, the fold expansion is determined by the priming alone, and ***Y*** = ***C*** ^***(prime)***^. We consider two possible ways to include the STAT/NF‐κB contributions. (1) *Linear contribution in priming* (*linear‐priming*): Here the contributions of STAT/NF‐κB arise as a sum of linear terms of {S_p_} such that *Y*_*α*_ = ∑_p_ [*a*_p_[S_p_^(prime)^]_α_], where the index α represents the cytokine cocktail condition. This leads to five predictor variables in the design matrix **X** in the presence of activated STAT1 (S1), STAT3 (S3), STAT4 (S4), STAT5 (S5) and NF‐κB (Sb). (2) *Linear + pairwise contribution* (*linear + pairwise priming*): The contribution is constructed with linear and pairwise STAT and NF‐κB terms, present in the priming stage in the presence of S1, S3, S4, S5, Sb and their all possible cross terms: *Y*_*α*_ = ∑_p_ [*a*_p_ [S_p_^(prime)^]_α_ + ∑_q,p ≠ q_ *b*_pq_ [S_p_^(prime)^]_α_[S_q_^(prime)^]_α_]. This leads to 15 predictor variables in the design matrix **X**.
2. ***Post‐priming alone***: Here, the fold expansion in NK cells is determined by the post‐priming (PP‐I) alone, and ***Y = C*** ^**(PP*‐*I)**^. We consider two models. (1) *Linear contribution (linear‐PP‐I)*: Contains sum of linear STAT/NF‐κB terms that is, *Y*_*α*_ *=* ∑_p_ *c*_p_ [S_p_^(PP‐I)^]_α_. This leads to five predictor variables in the design matrix **X**. (2) *Linear + pairwise contribution (linear + pairwise‐PP‐I)*: contains linear and pairwise STAT/NF‐κB terms. Thus, *Y*_*α*_ *=* ∑_p_ [*c*_p_ [S_p_^(PP‐I)^]_α_ + ∑_q,p ≠ q_ *d*_pq_[S_p_^(PP‐I)^]_α_[S_q_^(PP‐I)^]_α_]. This leads to 15 predictor variables in the design matrix **X**.
3. ***Additive contribution***: In this case, the NK cell fold expansion is given by ***Y = C*** ^***(prime)***^ ***+ C*** ^***(PP‐I)***^. We consider two models. (1) *Linear contributions from the priming and the PP‐I stage (linear priming* ⊕ *linear–PP‐I)*: Here the regression is given by *Y*_*α*_ = ∑_p_ [*a*_p_ [S_p_^(prime)^]_α_ + *c*_p_[S_p_^(PP‐I)^]_α_]. This leads to 10 predictor variables in the design matrix **X**. (2) *Linear + pairwise contributions from the priming and the PP‐I stage (linear + pairwise priming* ⊕ *linear + pairwise–PP‐I)*: The regression model is given by *Y*_*α*_ = ∑_p_ [*a*_p_[S_p_^(prime)^]_α_ + ∑_q,p ≠ q_ *b*_pq_[S_p_^(prime)^]_α_[S_q_^(prime)^]_α_] *+* ∑_p_ [*c*_p_[S_p_^(PP‐I)^]_α_ + ∑_q,p ≠ q_ *d*_pq_[S_p_^(PP‐I)^]_α_[S_q_^(PP‐I)^]_α_]. This leads to 30 predictor variables in the design matrix **X**.
4. ***Multiplicative contribution***: In this case, the NK cell fold expansion is given by *Y*_*α*_ = C^(prime)^_α_C^(PP‐I)^_α_. We consider two models. (1) *Pairwise contributions from the priming and the PP‐I stage (linear priming* ⊗ *linear–PP‐I)*: Here the regression model is given by *Y*_*α*_ *=* (∑_p_ *a*_p_[S_p_^(prime)^]_α_) × (∑_p_ *c*_p_[S_p_^(PP‐I)^]_α_). This leads to 25 predictor variables in the design matrix **X**. (2) *Pairwise + higher‐order contributions from the priming and the PP‐I period (linear + pairwise‐priming* ⊗ *linear + pairwise–PP‐I)*: The regression here is given by *Y*_*α*_ = (∑_p_ [*a*_p_[S_p_^(prime)^]_α_ + ∑_q,p ≠ q_ *b*_pq_[S_p_^(prime)^]_α_[S_q_^(prime)^]_α_]) × (∑_p_[*c*_p_[S_p_^(PP‐I)^]_α_ + ∑_q,p ≠ q_ *d*_pq_[S_p_^(PP‐I)^]_α_[S_q_^(PP‐I)^]_α_]). This leads to 225 predictor variables in the design matrix **X**.

We group unique STAT/NF‐κB contributions, represented by unique terms on the right‐hand sides of the aforesaid regressions, by the parameters *β*_*k*_ and explanatory (predictor) binary variables *X*_*k*_, denoting *S*_*p*_ or the product of several *S*_*p*_, and estimate the parameters *β*_*k*_ using LASSO regression (Supplementary table 4). *β* > 0 represents the synergy between pair‐wise STATs and NF‐κB, and *β* < 0 illustrates antagonism.

#### Calculation of linear regression coefficients (*β*) to quantify synergy or antagonism for our optimal map and regression model

Our investigation showed that imputation map 28 along with *linear priming* ⊗ *linear–PP‐I* regression model generates the best predictive model for the NK cell fold expansion on day 9. Here we evaluate the errors in the regression coefficients for the aforesaid map and the model. The regression coefficients can be used to interpret relevant STAT–NF‐κB synergy/antagonism that regulates the weighted averaged NK cell fold expansion across donors (***Y*** in Supplementary table 1b). First, we chose all 12 treatment cytokine conditions (Figure 1b) and applied our best predictive model and map (design matrix ***X*** of size 12 × 25) on the weighted average (***Y***) (Supplementary table 1b) to evaluate the regression coefficients of vector ***β*** of STAT–STAT or STAT–NF‐κB interactions between priming and PP‐I period. Using L1 regularization, we selected the predictor variables whose coefficients were nonzero. We found 21 predictor variables as important (nonzero values) among 25 and discarded the predictor variables whose coefficients were zero in the design matrix ***X***.

We computed the fluctuations in the ***β*** coefficients using a bootstrapping method, where we statistically generated *n* (=1000) bootstrap samples of the weighted average of NK cell fold expansion ***Y***_***Bootstrap***_ ≡ $\tilde{Y}$, each containing fold expansions for 12 cytokine conditions. To do that, we randomly selected *N*_*A*_ (=10) donors from the donor 1 (D1) to donor 10 (D10) for treatment conditions 1–6 (Supplementary table 1a). Then, we calculated variances (*σ*_i_^2^) in NK cell fold expansion across conditions 1–6 for each of these *N*_*A*_ donors and calculated weight ω_i_ (*i* = *1,…,N*_*A*_) for each of them based on their deviances (deviance_i_) as discussed in the Methods section *Calculating Weighted average (Y) for NK cell fold expansion*. Next, we calculated the weighted average of the NK cell fold expansion,^51^ for cytokine condition *α,* as ${\tilde{Y}}_{A}^{a}=\sum_{i=1}^{N_{A}}\omega_{i}{f_{i}}^{a}$, where $f_{i}^{\alpha }$ represents the fold expansion for *i*th donor treated with *α*th cytokine condition (Supplementary table 1a). Following the aforesaid method, we created ${\tilde{Y}}_{B}$ (of size 2 × 1) for cytokine cocktail treatment conditions 7 and 8 by selecting *N*_*B*_ (= 4) donors randomly from donors 11‐14. Similarly, we generated ${\tilde{Y}}_{C}$ (of size 4 × 1) for cytokine cocktail treatment conditions 9–12 and by selecting *N*_*C*_ (= 3) donors randomly from donors 15‐17. Next, we combined ${\tilde{Y}}_{A}$, ${\tilde{Y}}_{B}$ and ${\tilde{Y}}_{C}$ to obtain a bootstrapped weighted average of fold expansion $\tilde{Y}$ (of size 12 × 1) for all observed cytokine cocktail conditions. We used ***X*** (of size 12 × 21) and the bootstrapped weighted average $\tilde{Y}$ to calculate ***β*** (of size 21 × 1). We repeated this for *n* times and calculated ***β***_***j***_ each time (j = *1, 2,…,n*). We then evaluated the ratio of mean (< ***β*** >) and standard deviation (***σ***_***β***_) corresponding to each of the predictor variables (here the number of predictor variables is 21) and interpreted the existence of synergy and antagonism for those predictor variables that followed |*< β >/σ*_*β*_ | > 2^26^ (Supplementary table 4).

#### Leave‐one‐out cross‐validation for model selection

Given the NK cell fold expansion for 12 cytokine cocktail treatments, we find the overall cross‐validation error by dividing the data into training data of size 11 (that includes 11 cytokine conditions) and test data of size 1 (the excluded 1 cytokine condition; Figure 1b). We use multiple linear regression (with LASSO or, Ridge regularizations)^26^ to predict the NK cell fold expansion *Ŷ*_*α*_ for *α*th cytokine cocktail treatment condition. The total prediction error is calculated by summing over all 12 test sets, each representing an observed condition (Figure 1b): RSS = ∑_α = 1,…,12_ (*Y*_*α*_−*Ŷ*_*α*_)^2^. To predict the optimal cytokine condition for typical responders, we selected the imputation map that produces the minimum RSS (Figure 3d, e, Supplementary table 3 and Supplementary figure 1).

#### Calculation of R^2^ to quantify the performance of model prediction across 12 cytokine cocktail conditions

We have the weighted average of fold expansion (***Y***) across donors for 12 conditions (see Figure 1b and Supplementary table 1a). By performing leave‐one‐out cross‐validation, we predicted the weighted fold expansion (*Ŷ*_*α*_) for each condition *α*, where, *α* = 1, 2, …, 12. Then, for each combination of imputation map and regression model, we calculated *R* ^2^ between the true (***Y***) and the predicted values (***Ŷ***) of the weighted average of fold expansion from all 12 conditions by obtaining Pearson correlation coefficient (*ρ*), such that *R* ^2^ = *ρ* ^2^. Comparisons between the aforementioned models for leave‐one‐out cross‐validation in terms of RSS are shown in Supplementary figure 1 and Supplementary table 3.

#### RRelief correctly chooses the ground truth model

We applied RRelief to short‐list imputation maps that are consistent with the data. Here, we performed a sanity check for the RRelief algorithm,^27^ where we generated synthetic data from a ground truth model and evaluated whether RRelief was able to select the ground truth model.

We created a ground truth model of the weighted average of NK cell fold expansion $Y_{\mathrm{GT}}=X\hat{\beta }$, where the regression coefficients {${\hat{\beta }}_{j}$} are estimated using the design matrix ***X*** for the optimal map (map 28) and the model (*linear‐priming* ⊗ *linear‐PP‐I* model) and the weighted average (*Y*) of fold expansion data (of size 12 × 1) (see Supplementary table 1b). *Y*_*GT*_ provides synthetic data for the weighted average of the fold expansion for 12 treatment conditions. Next, we applied RRelief analysis on *Y*_*GT*_ to evaluate the weights for each of STATs/NF‐κB for the priming and PP‐I period by varying the design matrix ***X*** for 64 imputation maps (Supplementary table 2). This was carried out with constraints that were used in our map selection process with the experimental data (see Figure 3a). The constraints are the following: (i) cytokine cocktail treatment condition 2 activates the STAT3 and (ii) for NK cell expansion, the activation of STAT3 and NF‐κB plays a positive role (considered positive scores in RRelief).

- By varying 64 imputation maps, we calculated 64 weight vectors *W* from *Y*_*GT*_ *= MW*, where *M* is a matrix of size 12 × 10. Each row of *M* represents a cytokine cocktail condition. The first and last five columns represent the presence of STAT1, STAT3, STAT4, STAT5 and NF‐κB in the priming and in the PP‐I period, respectively. The elements of matrix *M* change with the imputation maps (Supplementary table 2).
- Each weight vector *W* (of size 10 × 1) gives ten scores [−1, +1] related to each feature variables (STAT and NF‐κB). We calculated the weights for each imputation map by varying the nearest neighbor number (*k*).

Finally, we selected the imputation maps that show positive scores for STAT3 and NF‐κB in both the priming and PP‐I period by varying the nearest neighbor number (*k* = 2,3, …, 10). This filtering method produced map 28 (ground truth model) along with other imputation maps 8, 14, 16, 18, 32, 42, 44, 58 and 60. This provides a sanity check on the RRelief method and indicates that our best predictive model (***X***) constructed with imputation map 28 captures the effect of STAT3 and NF‐κB inhibitors on fold expansion (see Figure 3a).

#### Prediction of a new cytokine condition by the optimal *in silico* model

To address the novelty of the model, we predicted the typical fold expansion for a new condition (condition 13) on day 9. We created a new condition (condition 13) to evaluate the effect of IL‐21 in the post‐priming phase of condition 3. Condition 13 contains IL‐12 + 15 + 18 + 21 in the priming period (0–16 h) and IL‐2 + 21 in the PP‐I period (16 h to 3 days). Our model predicted that the typical fold expansion decreases roughly by half (~11.75) in condition 13 compared with condition 3 (~20; Supplementary figure 7). This suggests that the presence of IL‐21 in post‐priming period I is not beneficial to proliferation as opposed to its presence in the priming period.

#### Calculation of statistical significance in the weighted average of fold expansion across cytokine conditions

Fold expansion data on day 8 are imputed as an average of the fold expansion data observed on day 7 and day 9. As NK cells start to die after day 9, we chose the imputed data on day 8 for statistical analysis. Fold expansion for each condition is calculated in terms of a weighted average across 10 donors (D1–D10). For a cytokine condition (e.g. condition 3), the weight for each donor is calculated based on two different weights: (1) viability weights and (2) variability weights using the fold expansion data.

The viability weight (*W*_viability_) for each donor is assigned based on the fold expansion at early timepoints (days 2 and 4) with the following criteria: (1) If the fold expansion at days 2 and 4 is greater than 1 and there is a consistent increase (i.e. fold expansion on day 4 > fold expansion on day 2), the weight is equal to the fold expansion at day 4. (2) If the fold expansion is greater than 1 but declines (i.e. fold expansion on day 4 < fold expansion on day 2), the weight is the average of the fold expansions on days 2 and 4. (3) For donors with fold expansion less than 1, the weight is the minimum fold expansion between day 2 and day 4. Finally, the viability weights across all donors (D1–D10) for each cytokine condition are normalized so that they sum to 1.

To calculate the variability weight (*W*_variability_) for donors, we examined the extreme fold expansion values (outliers) of the donors for each cytokine condition. We calculated the normalized variability weight for the *i*th donor as *W*_variability_^i^ = (1/*d*_*i*_)/∑_I_ = (1/*d*_*i*_), where *d*_*i*_ = (fe_*i*_‐median({fe_*j*_, *j* ≠ *i*}))^2^ represents deviance for the *i*th donor with fold expansion fe_*i*_ (here *i* = 1, …, 10).

After evaluating the cytokine condition‐specific normalized *W*_viability_ and *W*_variability_, we calculated a net weight given by *W*_net_ = (*W*_viability_ + *W*_variability_). The net weights for donors within a condition were then renormalized so that the sum of the normalized *W*_net_ equals unity. The normalized *W*_net_ was used to compute the weighted average of fold expansion for a condition.

Next, we bootstrapped the fold expansion data for donors across cytokine conditions and recalculated the donor weights and the weighted average of fold expansion 10000 times to generate distributions of the weighted average of fold expansion for conditions 1–6 (Figure 3i, left). By performing Fisher's least significant difference test,^29^ we verified that the weighted averages for conditions 1–6 are unequal (*P* < 0.005). We then performed the permutation test^30^ between pair‐wise conditions, specifically comparing condition 3 with the others. Condition 3 showed a significantly higher weighted average (*P* < 0.05) than conditions 1, 2 and 4–6 within the same‐group donors, with statistical significance *P* < 0.05 (see Figure 3i, right).

## AUTHOR CONTRIBUTIONS

**Indrani Nayak:** Formal analysis; investigation; methodology; software; validation; visualization; writing – original draft; writing – review and editing. **Rosalba Biondo:** Formal analysis; investigation; methodology; software; validation; visualization; writing – original draft; writing – review and editing. **William C Stewart:** Methodology. **Rebecca J Fulton:** Investigation. **Nina Möker:** Writing – review and editing. **Congcong Zhang:** Writing – review and editing. **Salim I Khakoo:** Conceptualization; funding acquisition; investigation; methodology; project administration; supervision; writing – original draft; writing – review and editing. **Jayajit Das:** Conceptualization; funding acquisition; investigation; methodology; project administration; supervision; writing – original draft; writing – review and editing.

## CONFLICT OF INTEREST

The authors declare no conflicts of interest.

## CODE AVAILABILITY

All the codes are written in MATLAB (The MathWorks Inc., R2020b, version 9.9) software. Codes describing our *in silico* models are available at the link https://github.com/indraniny/Optimal_cytokine_combo_project.

## Supporting information


Supplementary figure 1
Supplementary figure 2
Supplementary figure 3
Supplementary figure 4
Supplementary figure 5
Supplementary figure 6
Supplementary figure 7
Supplementary figure 8
Supplementary table 1
Supplementary table 2
Supplementary table 3
Supplementary table 4
Supplementary table 5

## Data Availability

Raw data files of NK cell proliferation that support the findings of this study are openly available at the following link: https://github.com/indraniny/Optimal_cytokine_combo_project/tree/main/data. Further data are available from the corresponding authors upon request, except for identifying donor information.
