# Supplementary material for: Modeling the response to interleukin‐21 to inform natural killer cell immunotherapy
Source: Immunol Cell Biol. 2025 Jan 25;103(2):192–212. doi: 10.1111/imcb.12848 (PMC11792776; doi:10.1111/imcb.12848)
Supplement: Supplementary file 1 — Supplementary figure 1 Supplementary figure 2 Supplementary figure 3 Supplementary figure 4 Supplementary figure 5 Supplementary figure 6 Supplementary figure 7 Supplementary figure 8 Supplementary table 1 Supplementary table 2 Supplementary table 3 Supplementary table 4 Supplementary table 5 [file IMCB-103-192-s001.docx]

Supplementary Materials for

**Modelling the response to IL-21 to inform NK cell immunotherapy**

Indrani Nayak^*^, Rosalba Biondo^*^, William C Stewart, Rebecca J Fulton, Nina Möker, Congcong Zhang, , Salim I Khakoo^+^, Jayajit Das^+^

**Supplementary Figures**

**

**Supplementary Figure 1, related to Figure 3d.** **Comparison of prediction errors to choose the optimal map and regression model.** RSS (Residual Sum of Squares) as a measure of prediction error in weighted average of NK fold expansion (see ***Y*** in Supplementary Tables **1 and 3**) for various regularized regression models. Height of each bar represents RSS across 12 LOOCV test sets, each representing a cytokine cocktail condition (Figure **1b** in main text). Light and dark green colors represent the regression model with LASSO (L1) and Ridge (L2) regularization, respectively (26). The left most regression model *(linear-priming* $\otimes$*linear-PP-I)* with L1 regularization (light green) gives the minimum RSS, or the best prediction of fold expansion. **RSS shown here, for each regularized regression model, are estimated for the optimal imputation map (varying maps 8, 14, 16, 28, 42, 44 to find the minimum RSS, See Figure **3d** in main text). For Ridge or Lasso regularization, the regularization constant λ is varied (λ = 1e-11,1e-6,1e-3,…,1.0) to choose an optimal λ that gives minimum RSS.

Supplementary Figure 2, related to Figure 5. Effect of IL-18 and IL-21 on NK cell fold expansion, cytotoxicity, and cell receptor expressions against hepatocellular carcinoma cell line HepG2. All data shown here are for high CD16 expressing donors. High and low CD16 are gated based on the median of CD16 mean expressions from 11 donors (D3-D10, D15-D17) treated with 10 cytokine conditions (conditions 1-6,9-12). (a) Percentage of CD107a at day 10 is plotted against the NK fold expansion at day 9. Data are labelled based on the treatment conditions in the presence of either IL-18 (red) or, IL-21 (blue) or, both IL-18+21 (green) or none (gray). (b) Percentage of CD107a expressions is plotted against absolute cell receptor expressions. Pearson correlations (ρ) and *P*-values are shown between %CD107a and each of absolute receptor expressions. NKp46, NKG2C and NKG2C show negative correlation with %CD107a.

Supplementary Figure 3, related to figure 5. Effect of IL-18 and IL-21 on NK cell fold expansion, cytotoxicity, and cell receptor expressions against hepatocellular carcinoma cell line PLC. All data shown here are for high CD16 expressing donors. High and low CD16 are gated based on the median of CD16 mean expressions from 11 donors (D3-D10, D15-D17) treated with 10 cytokine conditions (conditions 1-6,9-12). (a) Percentage of CD107a at day 10 is plotted against the NK fold expansion at day 9. Data are labelled based on the treatment conditions in the presence of either IL-18 (red) or, IL-21 (blue) or, both IL-18+21 (green) or none (gray). (b) Percentage of CD107a expressions is plotted against absolute cell receptor expressions. Pearson correlations (ρ) and *P*-values are shown between %CD107a and each of absolute receptor expressions. All receptor expressions show negative correlation with %CD107a.

Supplementary Figure 4, related to figure 5. Effect of IL-18 and IL-21 on NK cell fold expansion, cytotoxicity and cell receptor expressions hepatocellular carcinoma cell line SNU475. All data shown here are for high CD16 expressing donors. High and low CD16 are gated based on the median of CD16 mean expressions from 11 donors (D3-D10, D15-D17) treated with 10 cytokine conditions (conditions 1-6,9-12). (a) Percentage of CD107a at day 10 is plotted against the NK fold expansion at day 9. Data are labelled based on the treatment conditions in the presence of either IL-18 (red) or, IL-21 (blue) or, both IL-18+21 (green) or none (grey). (b) Percentage of CD107a expressions is plotted against absolute cell receptor expressions. Pearson correlations (ρ) and *P*-values are shown between %CD107a and each of absolute receptor expressions. NKp46, NKG2C and NKG2C expressions show negative correlation with %CD107a.


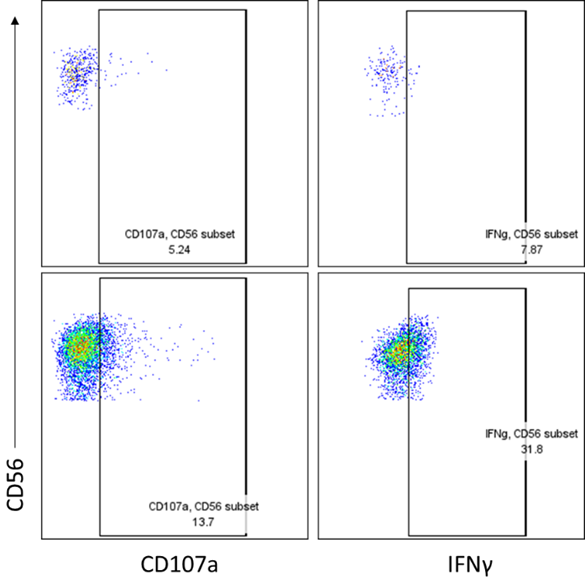


Supplementary Figure 5. Gating strategy to identify CD107a^+^ NK during degranulation, using flow cytometry. NK cells were isolated from PBMC and cultured with different cytokines combinations for 10 days. NK cells were incubated without target cells (NT= no target) to detect the baseline degranulation of NK cells. The same gating was applied when NK cells were incubated with target cells (HepG2, as shown by the representative plot. The same gating was applied to the other cell lines used as targets, PLC, SNU475, Huh7). The final CD107a^+^ NK cells with each target cell line were calculated by subtracting from it the NT CD107a^+^ NK. The same gating strategy was applied to all the donors used.


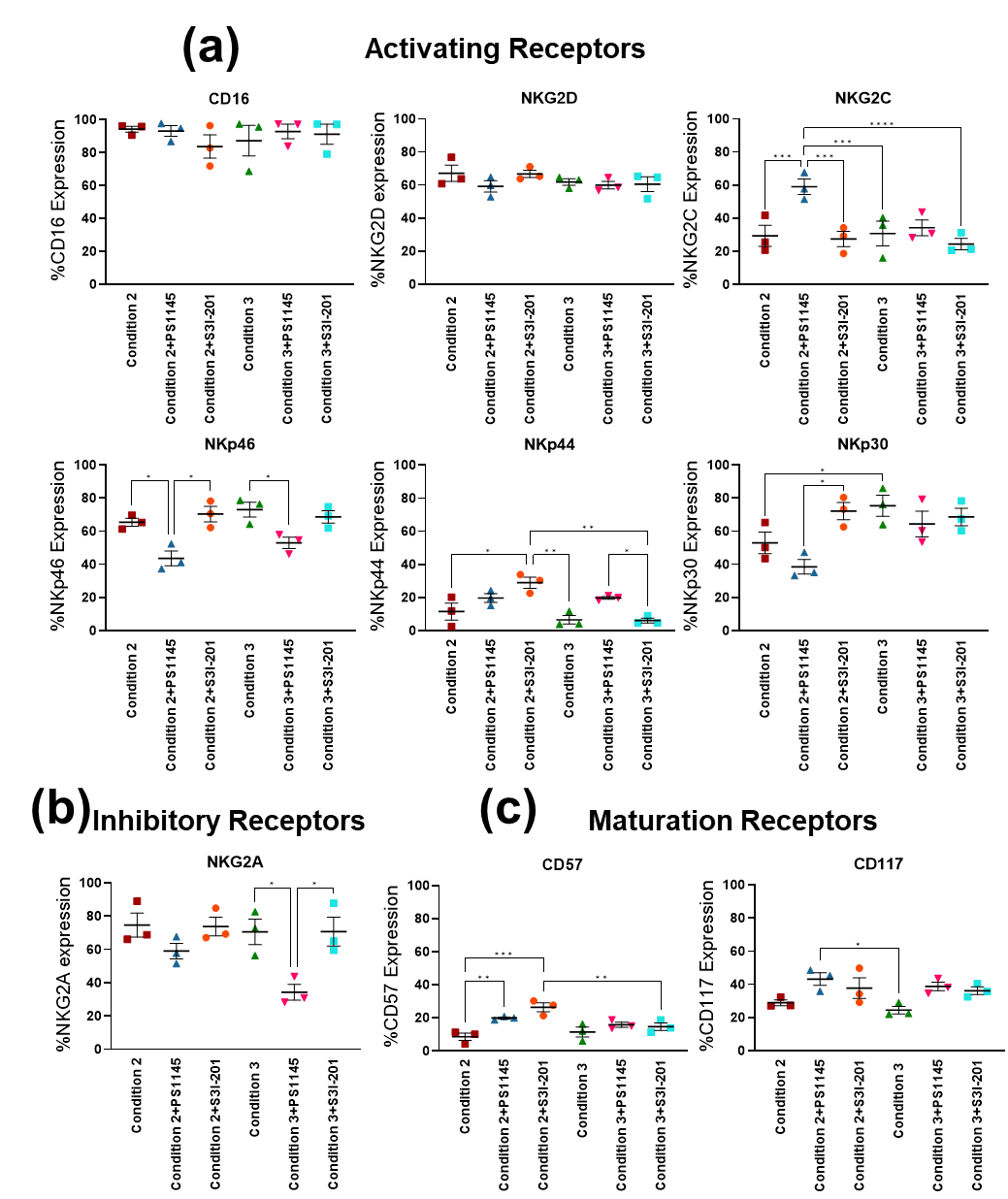


**Supplementary Figure 6, related to figure 4.** **Effect of STAT3 and NF-κB inhibition on the phenotype of NK cells. (a)** Activating, **(b)** inhibitory, **(c)** maturation receptors expressed at day 10 of *in vitro* culture by cytokine-activated NK cells, cultured in the presence of STAT3 and NF-κB inhibitor during the 16hr priming stage. Data shown as mean of 3 different donors ± SEM. Data analyzed by RM one-way ANOVA, and Tukey multiple comparison correction. (**P*-value ≤ 0.05, ** *P*-value ≤ 0.01, *** *P*-value ≤ 0.001, **** *P*-value ≤ 0.0001).

**Supplementary Figure 7. In-silico model prediction for an unknown cytokine condition.** Fold expansion in weighted average for conditions 1-13 for donors used in the main text (D1-D10, Figure **3f**) on day 9. In-silico model is trained with the weighted averages of experimental data for conditions 1-12 (maroon) and predicted fold expansion for condition 13 (gray).

Supplementary Figure 8. Flow cytometry gating strategy to identify NKG2C and NKG2A expressions by NK cells for a donor. NK cells were isolated from PBMC and cultured with different cytokine combinations for 10 days. CD56+ NK cells were analysed for the co-expression of NKG2C and NKG2A by flow cytometry. Untreated indicates freshly isolated NK cells with no cytokine. These cells were analysed by flow cytometry directly after isolation and were not kept in culture with cytokines. Representative example of gating strategy. The same gating strategy was applied to all the donors used.

**Supplementary Tables**

**Supplementary Table 1a: NK cell fold expansion across donors for various cytokine cocktail treatment conditions at day 9**

| **Donor**  **Treatment** | D1 | D2 | D3 | D4 | D5 | D6 | D7 | D8 | D9 | D10 |
| --- | --- | --- | --- | --- | --- | --- | --- | --- | --- | --- |
| Condition 1 | 1.45 | 3.15 | 23.4 | 23.4 | 7.96 | 7.7 | 5.8 | 6.34 | 26.07 | 13.74 |
| Condition 2 | 1.75 | 1.29 | 2.28 | 7.12 | 0.82 | 2.22 | 9 | 11.76 | 21.9 | 33.6 |
| Condition 3 | 2.5 | 3.63 | 4.06 | 23.2 | 0.46 | 10.38 | 69.84 | 22.71 | 18.3 | 21.99 |
| Condition 4 | 0.95 | 2.24 | 13.4 | 22 | 10.42 | 7.98 | 5.76 | 4.1 | 31.8 | 4.8 |
| Condition 5 | 1.24 | 2.65 | 19.24 | 15.18 | 1.04 | 9.68 | 8.18 | 6.18 | 15.6 | 8.13 |
| Condition 6 | 1.35 | 2.32 | 18.26 | 15.18 | 16.38 | 5.5 | 20.64 | 5.3 | 34.32 | 7.17 |

| **Donor**  **Treatment** | D11 | D12 | D13 | D14 |
| --- | --- | --- | --- | --- |
| Condition 7 | 10.2 | 12 | 4.68 | 9 |
| Condition 8 | 9.6 | 15 | 6 | 11.1 |

| **Donor**  **Treatment** | D15 | D16 | D17 |
| --- | --- | --- | --- |
| Condition 9 | 15 | 12 | 0.4 |
| Condition 10 | 18 | 14.4 | 16.8 |
| Condition 11 | 12 | 11.7 | 10.8 |
| Condition 12 | 13.8 | 15.6 | 16.8 |

**Supplementary Table 1b: Weighted and standard average of NK cell fold expansion across donors for various cytokine cocktail conditions**

|  | Weighted average (***Y***) | Standard average (***Y̅***) |
| --- | --- | --- |
| Condition 1 | 11.94 | 11.90 |
| Condition 2 | 13.63 | 9.17 |
| Condition 3 | 20.55 | 17.71 |
| Condition 4 | 11.85 | 10.34 |
| Condition 5 | 8.68 | 8.71 |
| Condition 6 | 13.35 | 12.64 |
| Condition 7 | 7.31 | 8.97 |
| Condition 8 | 8.80 | 10.42 |
| Condition 9 | 14.61 | 9.13 |
| Condition 10 | 17.55 | 16.40 |
| Condition 11 | 11.96 | 11.50 |
| Condition 12 | 14.03 | 15.40 |

**Supplementary Table 2: Possible imputation Maps or induced STATs/NF-κB transcription factors by Interleukins used in our in-silico model**

| **Imputation Map**  **Index** | **IL-2** | **IL-12** | **IL-15** | **IL-18** | **IL-21** |
| --- | --- | --- | --- | --- | --- |
| 1 | STAT5 | STAT4 | STAT5 | NF-κB | STAT3 |
| 2 | STAT5 | STAT4 | STAT5 | NF-κB, STAT3 | STAT3 |
| 3 | STAT5 | STAT4 | STAT5 | NF-κB | STAT3, STAT1 |
| 4 | STAT5 | STAT4 | STAT5 | NF-κB, STAT3 | STAT3, STAT1 |
| 5 | STAT5, STAT1 | STAT4 | STAT5 | NF-κB | STAT3 |
| 6 | STAT5, STAT1 | STAT4 | STAT5 | NF-κB, STAT3 | STAT3 |
| 7 | STAT5, STAT1 | STAT4 | STAT5 | NF-κB | STAT3, STAT1 |
| 8 | STAT5, STAT1 | STAT4 | STAT5 | NF-κB, STAT3 | STAT3, STAT1 |
| 9 | STAT5, STAT3 | STAT4 | STAT5 | NF-κB | STAT3 |
| 10 | STAT5, STAT3 | STAT4 | STAT5 | NF-κB, STAT3 | STAT3 |
| 11 | STAT5, STAT3 | STAT4 | STAT5 | NF-κB | STAT3, STAT1 |
| 12 | STAT5, STAT3 | STAT4 | STAT5 | NF-κB, STAT3 | STAT3, STAT1 |
| 13 | STAT5, STAT4 | STAT4 | STAT5 | NF-κB | STAT3 |
| 14 | STAT5, STAT4 | STAT4 | STAT5 | NF-κB, STAT3 | STAT3 |
| 15 | STAT5, STAT4 | STAT4 | STAT5 | NF-κB | STAT3, STAT1 |
| 16 | STAT5, STAT4 | STAT4 | STAT5 | NF-κB, STAT3 | STAT3, STAT1 |
| 17 | STAT5, NF-κB | STAT4 | STAT5 | NF-κB | STAT3 |
| 18 | STAT5, NF-κB | STAT4 | STAT5 | NF-κB, STAT3 | STAT3 |
| 19 | STAT5, NF-κB | STAT4 | STAT5 | NF-κB | STAT3, STAT1 |
| 20 | STAT5, NF-κB | STAT4 | STAT5 | NF-κB, STAT3 | STAT3, STAT1 |
| 21 | STAT5, STAT3, STAT1 | STAT4 | STAT5 | NF-κB | STAT3 |
| 22 | STAT5, STAT3, STAT1 | STAT4 | STAT5 | NF-κB, STAT3 | STAT3, |
| 23 | STAT5, STAT3, STAT1 | STAT4 | STAT5 | NF-κB | STAT3, STAT1 |
| 24 | STAT5, STAT3, STAT1 | STAT4 | STAT5 | NF-κB, STAT3 | STAT3, STAT1 |
| 25 | STAT5, STAT4, STAT1 | STAT4 | STAT5 | NF-κB | STAT3 |
| 26 | STAT5, STAT4, STAT1 | STAT4 | STAT5 | NF-κB, STAT3 | STAT3 |
| 27 | STAT5, STAT4, STAT1 | STAT4 | STAT5 | NF-κB | STAT3, STAT1 |
| 28 | STAT5, STAT4, STAT1 | STAT4 | STAT5 | NF-κB, STAT3 | STAT3, STAT1 |
| 29 | STAT5, NF-κB, STAT1 | STAT4 | STAT5 | NF-κB | STAT3 |
| 30 | STAT5, NF-κB, STAT1 | STAT4 | STAT5 | NF-κB, STAT3 | STAT3 |
| 31 | STAT5, NF-κB, STAT1 | STAT4 | STAT5 | NF-κB | STAT3, STAT1 |
| 32 | STAT5, NF-κB, STAT1 | STAT4 | STAT5 | NF-κB, STAT3 | STAT3, STAT1 |
| 33 | STAT5, STAT4, STAT3 | STAT4 | STAT5 | NF-κB | STAT3 |
| 34 | STAT5, STAT4, STAT3 | STAT4 | STAT5 | NF-κB, STAT3 | STAT3 |
| 35 | STAT5, STAT4, STAT3 | STAT4 | STAT5 | NF-κB | STAT3, STAT1 |
| 36 | STAT5, STAT4, STAT3 | STAT4 | STAT5 | NF-κB, STAT3 | STAT3, STAT1 |
| 37 | STAT5, STAT3, NF-κB | STAT4 | STAT5 | NF-κB | STAT3 |
| 38 | STAT5, STAT3, NF-κB | STAT4 | STAT5 | NF-κB, STAT3 | STAT3 |
| 39 | STAT5, STAT3, NF-κB | STAT4 | STAT5 | NF-κB | STAT3, STAT1 |
| 40 | STAT5, STAT3, NF-κB | STAT4 | STAT5 | NF-κB, STAT3 | STAT3, STAT1 |
| 41 | STAT5, STAT4, NF-κB | STAT4 | STAT5 | NF-κB | STAT3 |
| 42 | STAT5, STAT4, NF-κB | STAT4 | STAT5 | NF-κB, STAT3 | STAT3 |
| 43 | STAT5, STAT4, NF-κB | STAT4 | STAT5 | NF-κB | STAT3, STAT1 |
| 44 | STAT5, STAT4, NF-κB | STAT4 | STAT5 | NF-κB, STAT3 | STAT3, STAT1 |
| 45 | STAT5, STAT4, STAT3, STAT1 | STAT4 | STAT5 | NF-κB | STAT3 |
| 46 | STAT5, STAT4, STAT3, STAT1 | STAT4 | STAT5 | NF-κB, STAT3 | STAT3 |
| 47 | STAT5, STAT4, STAT3, STAT1 | STAT4 | STAT5 | NF-κB | STAT3, STAT1 |
| 48 | STAT5, STAT4, STAT3, STAT1 | STAT4 | STAT5 | NF-κB, STAT3 | STAT3, STAT1 |
| 49 | STAT5, STAT3, STAT1, NF-κB | STAT4 | STAT5 | NF-κB | STAT3 |
| 50 | STAT5, STAT3, STAT1, NF-κB | STAT4 | STAT5 | NF-κB, STAT3 | STAT3 |
| 51 | STAT5, STAT3, STAT1, NF-κB | STAT4 | STAT5 | NF-κB | STAT3, STAT1 |
| 52 | STAT5, STAT3, STAT1, NF-κB | STAT4 | STAT5 | NF-κB, STAT3 | STAT3, STAT1 |
| 53 | STAT5, STAT4, STAT3, NF-κB | STAT4 | STAT5 | NF-κB | STAT3 |
| 54 | STAT5, STAT4, STAT3, NF-κB | STAT4 | STAT5 | NF-κB, STAT3 | STAT3 |
| 55 | STAT5, STAT4, STAT3, NF-κB | STAT4 | STAT5 | NF-κB | STAT3, STAT1 |
| 56 | STAT5, STAT4, STAT3, NF-κB | STAT4 | STAT5 | NF-κB, STAT3 | STAT3, STAT1 |
| 57 | STAT5, STAT4, STAT1, NF-κB | STAT4 | STAT5 | NF-κB | STAT3 |
| 58 | STAT5, STAT4, STAT1, NF-κB | STAT4 | STAT5 | NF-κB, STAT3 | STAT3 |
| 59 | STAT5, STAT4, STAT1, NF-κB | STAT4 | STAT5 | NF-κB | STAT3, STAT1 |
| 60 | STAT5, STAT4, STAT1, NF-κB | STAT4 | STAT5 | NF-κB, STAT3 | STAT3, STAT1 |
| 61 | STAT5, STAT4, STAT1, NF-κB, STAT3 | STAT4 | STAT5 | NF-κB | STAT3 |
| 62 | STAT5, STAT4, STAT1, NF-κB, STAT3 | STAT4 | STAT5 | NF-κB, STAT3 | STAT3 |
| 63 | STAT5, STAT4, STAT1, NF-κB, STAT3 | STAT4 | STAT5 | NF-κB | STAT3, STAT1 |
| 64 | STAT5, STAT4, STAT1, NF-κB, STAT3 | STAT4 | STAT5 | NF-κB, STAT3 | STAT3, STAT1 |

**Supplementary Table 3: Comparison of RSS and overall fold expansion predictability of 12 cytokine cocktail conditions (R^2^) between regression models**

**a: LASSO regularization**

| **Design matrix for Regression Model** | **Optimal Imputation Map** | **Number of predictors (p) in design matrix X** | **R^2^** | **RSS** |
| --- | --- | --- | --- | --- |
| *linear-priming* $\otimes$*linear-PP-I* | 28 | 25 | 0.55 | 105 |
| *linear-priming* | 8 | 5 | 0.055 | 1176 |
| *linear-PP-I* | 8 | 5 | NAN | 2136 |
| *linear-priming* $\oplus$*linear-PP-I* | 8 | 10 | 0.01 | 1230 |
| *linear+pairwise-priming* | 42 | 15 | 0.022 | 286.744 |
| *linear+pairwise-PP-I* | 42 | 15 | 0.075 | 346.97 |
| *linear+pairwise-priming* $\oplus$*linear+pairwise-PP-I* | 42 | 30 | 0.029 | 456.56 |
| *linear+pairwise-priming* $\otimes$*linear+pairwise-PP-I* | 28 | 225 | 0.122 | 166.69 |

**b: Ridge regularization**

| **Design matrix for Regression Model** | **Optimal Imputation Map** | **Number of predictors (p) in design matrix X** | **R^2^** | **RSS** |
| --- | --- | --- | --- | --- |
| *linear-priming* $\otimes$*linear-PP-I* | 8 | 25 | 0.179 | 146.709 |
| *linear-priming* | 42 | 5 | 0.029 | 209.97 |
| *linear-PP-I* | 8 | 5 | 0.077 | 194.59 |
| *linear-priming* $\oplus$*linear-PP-I* | 8 | 10 | 0.001 | 194.93 |
| *linear+pairwise-priming* | 42 | 15 | 0.029 | 210.09 |
| *linear+pairwise-PP-I* | 8 | 15 | 0.02 | 202.94 |
| *linear+pairwise-priming* $\oplus$*linear+pairwise-PP-I* | 8 | 30 | 0.001 | 249.73 |
| *linear+pairwise-priming* $\otimes$*linear+pairwise-PP-I* | 8 | 225 | 0.026 | 138.009 |

**Supplementary Table 4: STAT-STAT and STAT-NF-κB synergy between priming and PP-I**

| Variable in Linear regression model | <β>/σ_β_ |
| --- | --- |
| **S_1_S̃_1_** | **2.2396** |
| S_1_S̃_3_ | -0.2233 |
| S_1_S̃_4_ | -1.9139 |
| **S_1_S̃_5_** | **2.6298** |
| S_1_S̃_b_ | -1.4216 |
| **S_3_S̃_1_** | **6.042** |
| S_3_S̃_3_ | 0.0385 |
| **S_3_S̃_4_** | **-2.3203** |
| S_3_S̃_5_ | 1.5327 |
| **S_3_S̃_b_** | **2.4948** |
| S_4_S̃_1_ | -1.5219 |
| **S_4_S̃_3_** | **-4.2445** |
| **S_4_S̃_4_** | **4.2445** |
| S_4_S̃_b_ | 1.3767 |
| S_5_S̃_3_ | 0.3846 |
| S_5_S̃_4_ | 1.0845 |
| S_5_S̃_b_ | 0.3916 |
| **S_b_S̃_1_** | **-2.4783** |
| S_b_S̃_3_ | 1.2028 |
| S_b_S̃_4_ | -1.2028 |
| S_b_S̃_b_ | 1.2028 |

** S_i_ and S̃_i_ represent induced STAT/NF-κB during the priming or PP-I period, respectively.

IL-21 in priming results in the activation of STAT3 and STAT1 in the priming and which contributes to the term **S_1_S̃_1,_** **S_1_S̃_5_** , **S_3_S̃_1_** , **S_3_S̃_4_** , **S_3_S̃_b_** significantly and synergistically (positive value) except the **S_3_S̃_4_** term (negative value). This quantification of synergy or antagonism might not reflect the true STAT or NF-κB interactions that regulate NK cell fold expansions.

**Supplementary Table 5: Correlation between %CD107a and NK receptor expressions against HCC cell lines**

| Cell lines | Receptors with positive Pearson correlation (ρ) with %CD107a | Receptors with negative Pearson correlation (ρ) with %CD107a | Correlation between NK cell fold expansion (day 9) and %CD107a expressions |
| --- | --- | --- | --- |
| Huh7 | NKp44, NKp30 | NKG2D, NKG2C, NKG2A | ρ = -0.29, *P*-value = 0.04 |
| HepG2 |  | NKp46, NKG2C, NKG2A | ρ = -0.11, *P*-value = 0.36 |
| PLC | NKG2D | NKp44, NKp30, NKp46,  NKG2C, NKG2A | ρ = -0.12, *P*-value = 0.34 |
| SNU475 |  | NKp46, NKG2C, NKG2A | ρ = -0.16, *P*-value = 0.20 |

** For the receptors in 2^nd^ and 3^rd^ column, Pearson Correlation (ρ) are associated with *P*-values < 0.05.
